# Elite squash players nutrition knowledge and influencing factors

**DOI:** 10.1186/s12970-021-00443-3

**Published:** 2021-06-10

**Authors:** Ollie Turner, Nigel Mitchell, Alan Ruddock, Alison Purvis, Mayur Ranchordas

**Affiliations:** 1 Academy of Sport & Physical Activity, Sheffield Hallam University, Sheffield, England; 2 English Institute of Sport, Sheffield, England

**Keywords:** Sports nutrition, Questionnaire, Racket sport, Individual sport

## Abstract

**Background:**

There is a reported mismatch between macronutrient consumption and contemporary macronutrient guidelines in elite standard squash players. Suboptimal dietary practices could be due to a lack of nutrition knowledge among players. Subsequently, the purpose of this study was to assess the sports nutrition knowledge of elite squash players through the Nutrition for Sport Knowledge Questionnaire (NSKQ) and provide an indication of whether players require nutrition support to increase their nutrition knowledge.

**Methods:**

This cross-sectional study assessed the nutrition knowledge of 77 elite squash players via the NSKQ over the period of June 2020 to August 2020.

**Results:**

Players conveyed average nutrition knowledge with a mean NSKQ score of 48.78 ± 10.06 (56.07% ± 11.56%). There were no significant differences in NSKQ score between male and female players (*p* = .532). There was found to be a weak positive association between world ranking and NSKQ score (*r* = .208) and age and NSKQ score (*r* = .281). Players who had a relevant undergraduate degree (e.g. BSc Sport & Exercise Science) had significantly greater NSKQ score than players with no relevant qualifications (*p* = .022). Players who consulted a sports nutritionist to obtain their main source of nutrition information were shown to have significantly greater knowledge than those who acquired knowledge from a sports scientist (*p* = .01) or the internet / social media (*p* = .007).

**Conclusions:**

Players should consult with a sports nutritionist to increase their sport nutrition knowledge. Future research should quantify the effectiveness of a nutritional education intervention at increasing nutrition knowledge in players.

**Supplementary Information:**

The online version contains supplementary material available at 10.1186/s12970-021-00443-3.

## Background

Squash is a high intensity intermittent sport [1] which is classified as one of the four major racket sports [2]. Elite male squash players are reported to exhibit a mean energy expenditure of 4933 ± 620 kJ.h^− 1^, mean heart rate of 92 ± 3% heart rate maximum and mean respiratory exchange ratio of 0.94 ± 0.06 throughout simulated match play [3], conveying the high intensity nature of the sport [1]. At elite standard, players are reported to train for more than 12 h per week, with squash-specific sessions such as pressure sessions and continuous rallies eliciting heart rates above 90% heart rate maximum [4, 5]. Due to the high energetic demands of elite squash, adequate energy intake is required to optimise health and physical performance [6, 7]. Subsequently, sports nutritionists have become an integral part of high-performance teams to help promote optimal nutrition practices. Despite this, there is a paucity of information regarding specific nutritional recommendations for squash, unlike in other racket [8] and high intensity intermittent sports [9]. This makes it difficult for practitioners working with elite squash players, as they have to make recommendations based on non-specific guidelines [10].

Ventura-Comes et al., (2019) [11] analysed the food habits of elite Spanish squash players using a food consumption frequency questionnaire and found that players under consumed carbohydrate-rich foods such as bread, potatoes, pasta and rice when compared to contemporary guidelines [10]. Low frequency of carbohydrate rich foods are likely to result in low carbohydrate intakes in relation to guidelines, reducing high intensity intermittent performance [10].

Mismatches between contemporary nutritional recommendations and players habitual nutritional practices suggest that elite squash players might lack the nutrition knowledge to have optimal dietary practices. An athlete’s nutrition knowledge is one modifiable determinants of dietary behaviour [12], with a weak positive correlation being reported between an athlete’s nutrition knowledge and their diet quality [13, 14]. The association between nutrition knowledge and dietary behaviour is multifaceted and influenced by many other individual and environmental factors such as hunger and appetite, taste and food preferences, beliefs, culture, experiences, self-efficacy, financial status, peers, sporting culture, access to food and cooking skills [12–15]. The evaluation of association between nutrition knowledge and diet quality is also complex due to a plethora of inadequately or partially validated instruments to assess nutrition knowledge [12, 13] and inappropriate tools to quantify dietary intake [13], such as 24-h dietary recalls and three-day self-reported food diaries, which have both been shown to poorly estimate micronutrient intake [16, 17]. Athletes energy requirements are also highly variable throughout macro-, meso- and microcycles, as many adopt a periodised training approach [18, 19] complicating the assessment process [20]. Despite only a weak association between an athlete’s nutrition knowledge and their diet quality, nutrition education interventions have been shown to increase athlete’s nutrition knowledge and lead to greater dietary behaviours [21], optimising physical performance [22]. Subsequently, increasing an athlete’s nutrition knowledge is of interest to sport nutrition practitioners as it might enhance athlete’s dietary practices [21] and athletic ability [22].

To date, no study has quantified the nutritional knowledge of elite squash players. Assessing the nutrition knowledge of elite squash players would help provide an indication of whether players require nutrition support and education to increase their nutrition knowledge and improve food choices to support high training and match demands, as well as a general healthy lifestyle. The main aim of the study was to assess the nutrition knowledge of elite squash players using the validated NSKQ [23, 24]. The Secondary aim of the study was to investigate the factors which may influence an elite squash players nutrition knowledge. Greater standards of education have been shown to positively influence athlete’s nutrition knowledge [25–30], while sex [26, 27, 31–49], playing ability [28, 30, 39, 44, 46, 50–54], age [27, 41, 44, 46, 55–57] and main source of nutrition knowledge [27, 54] have all been reported to have equivocal influences on athlete’s nutrition knowledge. Consequently, the study aimed to assess the association between age and world ranking on nutrition knowledge and quantify whether players standard of relevant education and main source of nutrition knowledge influence nutrition knowledge.

The final aim of the study was to survey what contemporary sports nutrition research elite squash players would like to see being conducted in the future. There are currently no specific nutritional guidelines for elite squash players. By surveying players, the aim was to ensure that all relevant nutrition research in elite squash is undertaken, specific to player’s needs. This data can then be used to create a nutritional education intervention which is bespoke to elite squash players.

## Methods

### Participants

The research was approved by an institutional ethics committee (ER23597808). All participants who volunteered provided informed consent with the study being conducted according to the principles of the 7th revision of the Declaration of Helsinki [58].

**Fig. 1:**
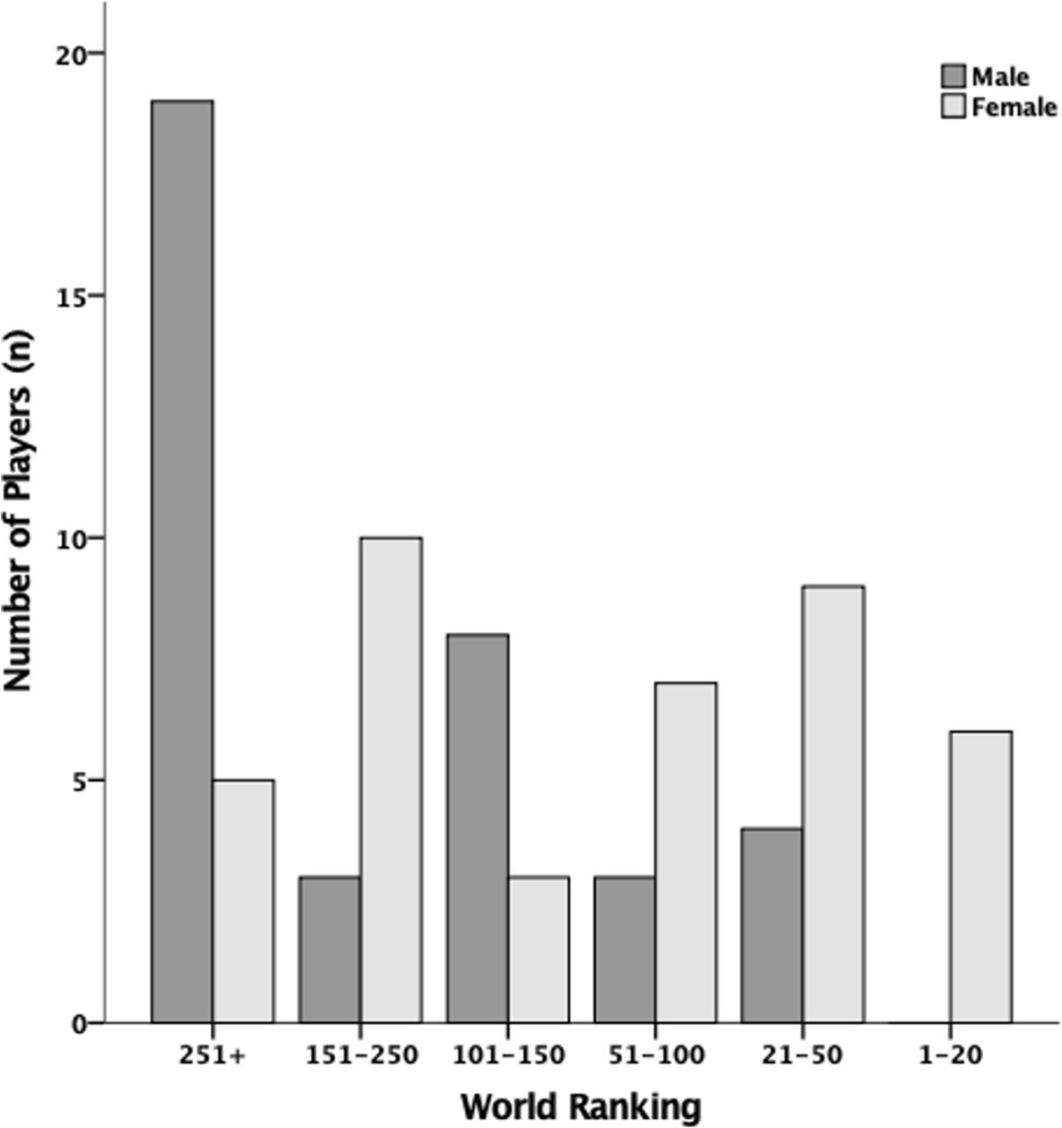
Distribution of player world ranking

A convenience sample of prospective participants were contacted through the Professional Squash Association (PSA) on two separate occasions (June 2020 and August 2020) and were provided with information about the study. Players were required to be a member of the PSA to take part in the study, with this forming the inclusion criteria for the study. Seventy-seven elite squash players took part in the study, 37 were male, and 40 were female. Responses were received from a global sample of the population (North America = 5; South America = 2; Europe = 55; Africa = 5; Asia = 6; and Oceania = 4). The mean average (± standard deviation) age and world ranking of the participants was 24 ± 5 and 190 ± 167 respectively. World rankings were taken from the PSA September rankings upon termination of the data collection period. Figure 1 details the distribution of the players world ranking.

### Nutrition knowledge

Players’ nutrition knowledge was measured via the validated NSKQ [23], using the revised version [24]. The NSKQ was chosen over other validated nutrition knowledge questionnaires as it is has ample applicability across a variety of cultures [23], is designed to be administered online [23], has a high construct validity [23] and underwent a comprehensive validation process utilising classical test theory [59] and Rasch analysis [60]. The questionnaire includes 87 questions with six subsections; weight management (*n* = 12), macronutrients (*n* = 30), micronutrients (*n* = 13), sports nutrition (*n* = 12), supplements (*n* = 12) and alcohol (*n* = 8). Nutrition knowledge was quantified using a scoring system set by Trakman et al. (2017) [23] of: poor (0–49%), average (50–65%), good (66–75%) and excellent (76–100%).

The NSKQ was written in English and was distributed online via Qualtrics (Washington, USA). Players were informed that the questionnaire would take approximately 25 min to complete [23] and instructed to complete the questionnaire in their own time without the use of resources (peers, books, internet etc.).

Two additional questions were asked upon completion of the NSKQ to quantify some of the factors influencing nutrition knowledge. Players were asked to ‘detail any relevant qualifications which are specific to nutrition’ (e.g. A-Level biology, BSc sport and exercise science etc.), as standard of education has been shown to positively influence nutrition knowledge [25–30]. These were subsequently ordered into four groups, taking the participants highest standard of relevant education (no qualification, A-Level [physical education / sport, biology, chemistry etc.] undergraduate degree [sport and exercise science, nutrition or equivalent] and postgraduate degree [sport and exercise science, nutrition or equivalent]). Players were also asked ‘where they obtained their main source of nutritional information from’ to gain an understanding of how many players currently consult with a sport nutritionist. Athletes have been shown to gain their nutrition information from a variety of sources including nutritionists, strength and conditioning coaches, sport specific coaches, peers, the internet etc. [29, 40, 48, 61–68]. Players were provided six options, with them selecting the most relevant (sports nutritionist; conditioning coach or sport scientist; squash coach; peer review journal articles; internet or social media; and other).

Finally, players were asked to share what squash nutrition research they would like to see in the near future. This was split into six options with players able to select their top three (quantification of energy expenditure throughout training periods in elite squash players to create specific nutritional training guidelines; quantification of energy expenditure throughout competition periods in elite squash players to create specific nutritional competition guidelines; quantification of sweat sodium losses in elite squash players to create specific hydration guidelines; nutrition to support immune function in elite squash players; efficacy of ergogenic aids in elite squash; other). These options were devised as they underpin the relevant knowledge to create specific nutritional recommendations for elite squash players (e.g. how much energy do elite squash players expend during training and competition, what are players sweat sodium losses etc.) and this data can be used to develop a nutritional education intervention specific to elite squash players.

### Statistical analysis

SPSS V 24.0 software (SPSS Inc., Chicago, IL) was used to perform the data analysis. All data was displayed as mean ± standard deviation for all participants with *p* < 0.05 being the criterion for significance among all statistical tests. The Kolmogrov-Smirnov test was used to check for normality. Levene’s Test for Equality of Variances was used to assess homogeneity of variance. Independent Samples T-Test or Mann-Whitney *U* Test (for non-parametric analysis) was used to analyse the differences in overall NSKQ scores and sub-section scores between male and female players. Effect sizes were interpreted according to accepted thresholds (small: *d* = 0.2; moderate: *d* = 0.5; large = 0.8) [69]. Pearson’s Correlation Coefficient or Spearman’s Rank-Order Correlation was used to quantify the relationship between NSKQ score or section scores against age and world ranking. Pearson’s Correlation Coefficient and Spearman’s Rank-Order Correlation were interpreted according to accepted thresholds (very weak: *r* = 0–0.19; weak: *r* = 0.2–0.39; moderate: *r* = 0.4–0.59; strong: *r* = 0.6–0.79; very strong: *r* = 0.8–1) [70].

A One-Way Analysis of Variance (One-Way ANOVA) or Kruskal-Wallis K Test (for non-parametric analysis) was performed to calculate any significant differences between NSKQ score or section scores against standard of relevant education and main source of nutrition information. Where a significant main effect was observed, the Hochberg Post-Hoc pairwise comparison was used to determine which groups were statistically significant.

## Results

### NSKQ score, subsection scores and individual question responses

**Fig. 2:**
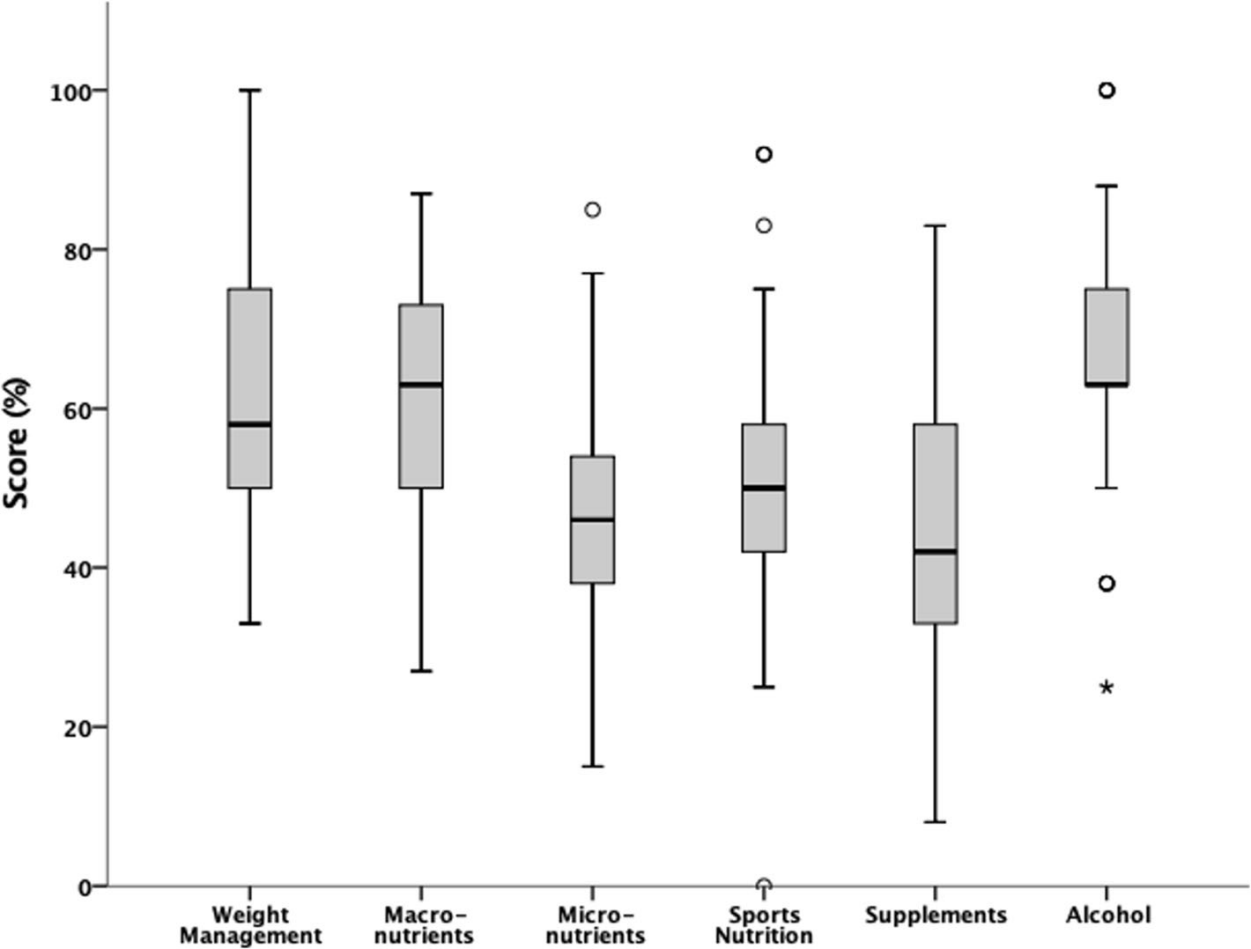
Players mean NSKQ subsection scores

The mean NSKQ score was 48.78 ± 10.06 (56.07% ± 11.56%), defined as “average” nutrition knowledge. Figure 2 details players grand mean subsection scores. The highest scoring section was alcohol, with players demonstrating “good” knowledge. Players had “average” macronutrient, weight management and sports nutrition knowledge. Supplements was the lowest scoring section with players exhibiting “poor” nutrition knowledge. Players also had “poor” knowledge of micronutrients. An additional file details individual question scores (see Supplementary Tables 1–87 for Individual Question Scores).

### Differences between male and female players in NSKQ score and subsection scores

**Fig. 3:**
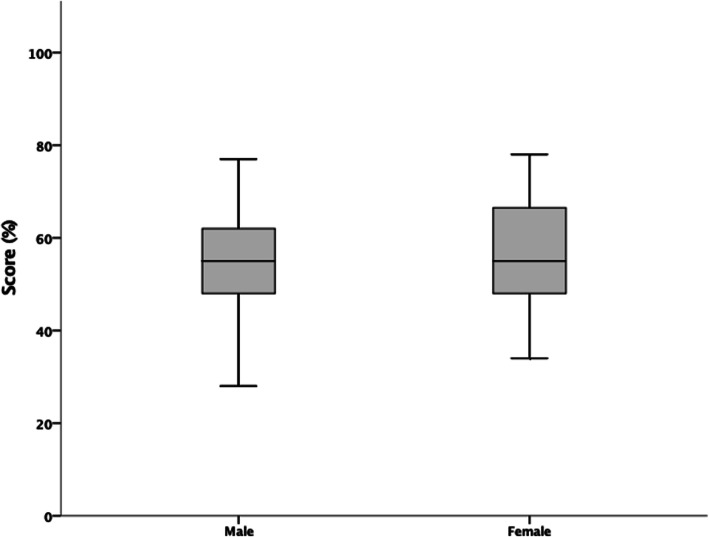
Differences between male and female players mean NSKQ scores

**Fig. 4:**
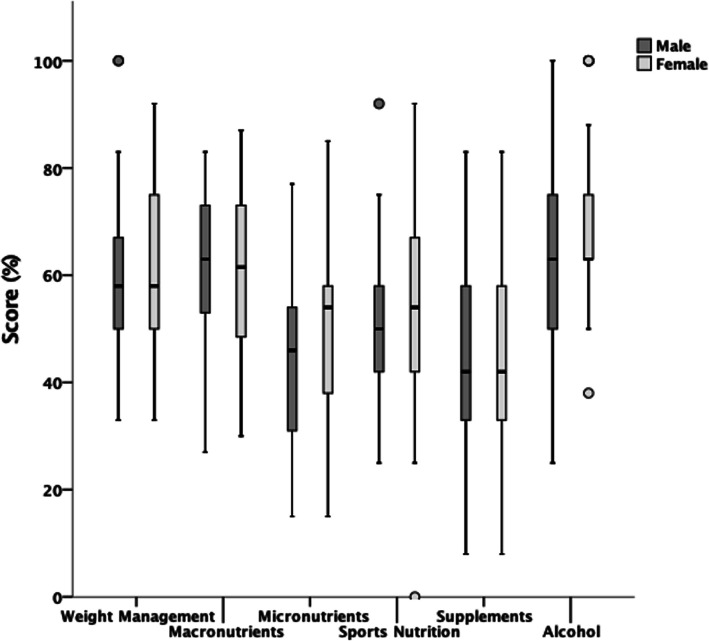
Differences between male and female mean NSKQ subsection scores

Figure 3 details differences between male and female players NSKQ score, with Fig. 4 detailing differences between male and female players subsection scores. There were no statistically significant differences between male and female players in NSKQ score or subsection scores (*p* > 0.05).

### Association between world ranking and NSKQ score and subsection score

World ranking had a weak positive association with NSKQ score (*r* = .208; *p* = .069) and weight management (*r* = .211; *p* = .066) subsection score. World ranking had a very weak positive association with macronutrient (*r* = .135; *p =* .241), micronutrient (*r* = .059; *p* = .610), sports nutrition (*r* = .137; *p* = .234), supplements (*r* = .154; *p* = .182) and alcohol (*r* = .170; *p* = .139) subsection scores.

### Association between age and NSKQ score and subsection score

Age had a weak positive association with NSKQ score (*r* = .281; *p* = .013), weight management (*r* = .288; *p* = .011), supplements (*r* = .255; *p* =) and alcohol (*r* = .215; *p* = .060) subsection scores. Age had a very weak positive association with macronutrient (*r* = .189; *p* = .099), micronutrient (*r* = .189; *p* = .100), and sports nutrition (*r* = .027; *p* = .813) subsection scores.

### Effects of standard of relevant education on NSKQ score

**Fig. 5:**
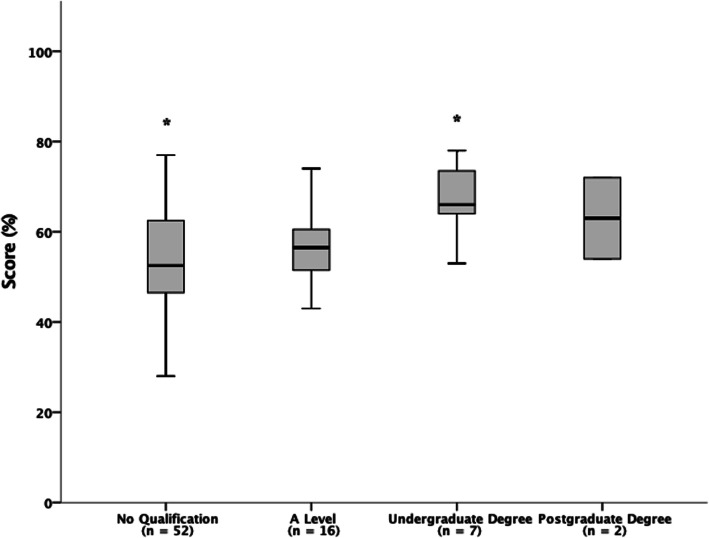
Effects of level of relevant education on RNSKQ score

Figure 5 details the effects of standard of relevant education on NSKQ score. Standard of education had a statistically significant effect on NSKQ score (*p* = .024). Hochberg Post-Hoc pairwise comparison revealed that players with a relevant undergraduate degree scored significantly better than players with no relevant qualification (*p* = .022; *d* = 1.3; CI = 1.15–22.09). No other statistically significant differences were observed across any of the groups when compared against each other.

### Effects of Main source of nutrition information on NSKQ score

**Fig. 6:**
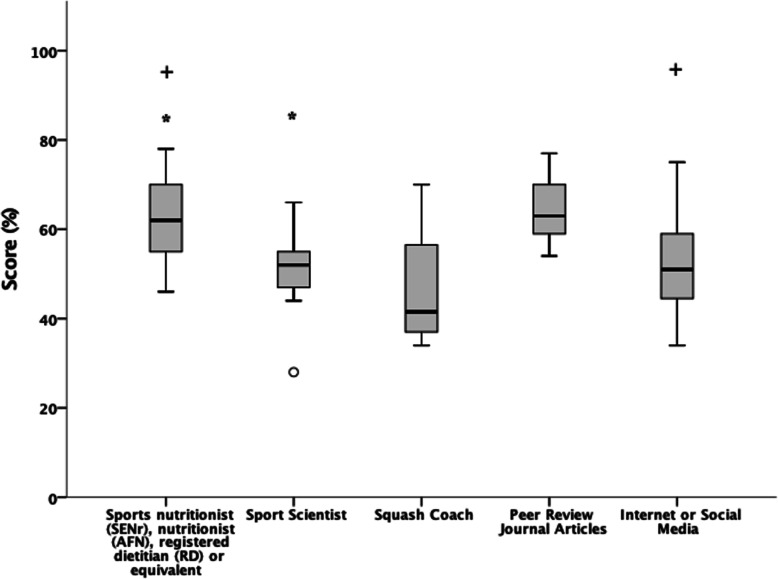
Effects of main source of nutrition information on RNSKQ score

Figure 6 details the effects of main source of nutrition information on NSKQ and subsection scores. Players main source of nutrition information had a significant positive effect on NSKQ score (*p* = .000). Hochberg Post-Hoc pairwise comparison showed that players who received their main source of nutrition information from a sport nutritionist, nutritionist, registered dietitian or equivalent scored significantly higher than players who received their main source of nutrition information from a sport scientist (*p* = .010; *d* = 1.2; CI = 1.61–18.62) or the internet / social media (*p* = .007; *d* = 0.96; CI = 1.57–15.62). No other significant differences were observed across any of the groups when compared against each other.

### Future sports nutrition research in elite squash

**Fig. 7:**
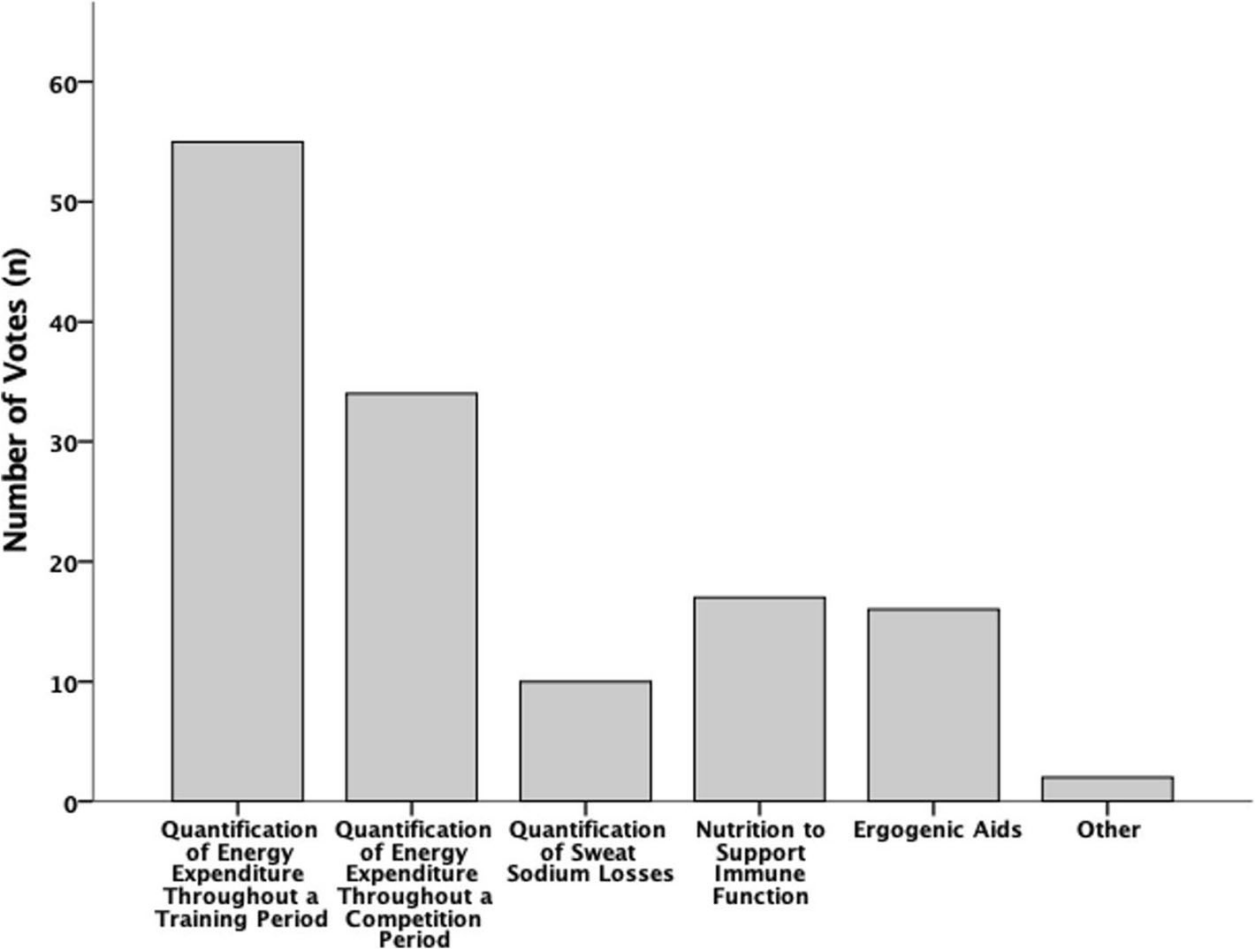
What sports nutrition research would elite squash players like to be conducted in the future

Figure 7 details players votes for future sports nutrition research in elite squash. The quantification of energy expenditure throughout training periods to create specific nutritional guidelines (*n* = 55; 71.43%) was the most popular area for future sports nutrition research in elite squash. The quantification of energy expenditure throughout competition periods to create specific nutritional competition guidelines was the second most popular (*n* = 34; 44.16%). Seventeen players stated they would like to see research regarding nutrition to support immune function (22.08%), with 16 players specifying they would like to see a quantification of ergogenic aids in squash (20.78%). The lowest scoring area of interest was the quantification of sweat sodium losses in squash to create specific nutritional guidelines (*n* = 10; 12.99%). Two players selected the ‘other’ suggesting “how to get everything into your diet while choosing plant based” and “the sustainability of a ketogenic diet for high performance in squash”.

## Discussion

The main aim of this study was to assess the nutrition knowledge of elite squash players. This study also aimed to quantify the factors which may influence an elite squash players nutrition knowledge. The final aim of the study was to survey what contemporary sports nutrition research elite squash players would like to see being conducted in the future.

The main findings of this study were (1) elite squash players had average nutrition knowledge, (2) there were no differences in nutrition knowledge between male and female players, (3) age and world ranking had a weak positive association with nutrition knowledge, (4) players who had a relevant undergraduate degree were found to have better nutrition knowledge than those who had no relevant qualification, (5) players who obtained their main source of nutrition knowledge from a sports nutritionist were shown to have better nutrition knowledge than players who obtained from a sports scientist or the internet, and (6) players valued quantifying the energetic demands throughout a training period as the research they would like to see undertaken in the future.

### Overall nutrition knowledge

Players nutrition knowledge was average (56%). Consequently, elite squash players should aim to increase their nutrition knowledge, as this may lead to greater dietary behaviours [21] and athletic ability [22]. Sport nutrition practitioners implement a variety of techniques to promote positive dietary behaviours, increasing nutrition knowledge in adolescent [47, 55, 71–76], collegiate [21, 22, 77, 78] and elite athletes [79]. Future research should aim to quantify the effectiveness of a nutritional education intervention at increasing elite squash players nutrition knowledge.

Evaluations of players nutrition knowledge in comparison to other athletes is difficult, due to the heterogeneity of tools used to assess nutrition knowledge [12, 80]. The NSKQ was devised as a universal tool to quantify athlete’s nutrition knowledge and make comparisons among different sports [23]. However, there are many other factors such as sex, playing ability, age, standard of education and main source of nutrition knowledge which influence an athlete’s nutrition knowledge [12] making comparisons challenging. To date, three previous studies [30, 65, 81] have used the NSKQ to assess sports nutrition knowledge. All three studies found athletes’ sports nutrition knowledge to be poor (Jenner et al., 2018 = 46% [65]; Trakman et al., 2018 = 46% [30]; McCrink et al., 2020 = 40% [81]) in contrast to the present findings. A plausible explanation for this is that the standard of athlete was higher in the present study in relation to the aforementioned studies [30, 65, 81]. This study featured six of the world’s top twenty squash players, and as a result these athletes may have greater access to resources (nutritionist, funding etc.) which may increase levels of nutrition knowledge [12]. Individual athletes such as elite squash players may also obtain personalised nutrition support which isn’t available to team sport athletes due to time and cost limitations. This personalised support can focus on the key concepts which need to be addressed specific to the athlete’s needs.

### Key concepts from individual questions

Players in the present study had poor knowledge of the contemporary carbohydrate and protein guidelines (see supplementary Tables 13 and 33 [10, 82]). This is consistent with findings from Ventura-Comes et al., (2019) [11] that elite Spanish squash players under consume carbohydrate in comparison to contemporary guidelines [10], with players rarely consuming foods which have a high carbohydrate content such as bread, potatoes, pasta and rice. Despite having poor knowledge of contemporary guidelines, players were able to identify the carbohydrate (see supplementary Tables 14–18) and protein (see supplementary Tables 32 and 42) content of foods, as well as appropriate protein sources to promote muscle growth post resistance training (see supplementary Tables 34–37). Players were also aware of what macronutrients to consume pre (see supplementary Table 59), during (see supplementary Table 64) and post exercise (see supplementary Table 66), as well as a suitable fuelling strategy (see supplementary Table 61) and snack during a 60–90-min session (see supplementary Table 65). This suggests that players may have greater procedural knowledge than declarative knowledge [83] and indicates that while players are unaware of contemporary nutrition guidelines, they understand the nutritional composition of foods along with the appropriate time to consume them, enabling them to follow optimal fuelling and recovery strategies. Many of the carbohydrate sources Ventura-Comes et al., (2019) [11] were reporting players to under consume were low glycemic index carbohydrates. It could be possible that squash players have a higher intake of high glycemic index carbohydrates. These are recommended around training sessions [10]. Future research should aim to quantify the training loads and energy expenditures of elite squash players to determine the nutritional requirements of the sport, as reported in other racket [8] and high intensity intermittent sports [9]. This would convey whether contemporary non-specific nutrition guidelines [10] are relevant to elite squash players, with this data informing any nutrition education intervention undertaken with elite squash players. Fifty five players (71%) surveyed in the present study conveyed that they would like to see this research undertaken. Players’ dietary intakes should also be quantified alongside training loads, as reported in other high intensity intermittent sports [84, 85]. This would give an insight into whether players’ dietary intake is optimal in relation to their training load [18, 19]. Food consumption frequency questionnaires, as reported by Venutra-Comes et al., (2019) [11] have been shown to display poor validity and reliability [86] in comparison to other methods such as weighed food diaries, snap ‘N’ send and 24-h dietary recall [17, 87]. Subsequently, more valid and reliable methods need to be employed to assess the energy intake and nutritional habits of elite squash players to obtain a better understanding of their dietary practices.

Players had poor micronutrient knowledge (45%). Quantifying players dietary intakes in future research would also provide an understanding of whether this lack of knowledge translates to poor diet quality. Tam et al., (2019) [80] reported that only 38% of nutritional education interventions include information regarding micronutrients. It is feasible that sport nutritionists focus on educating athletes to achieve an optimal diet specific to their needs, rather than educating athletes about micronutrient roles and requirements.

Players also had poor supplementation knowledge (45%). This is consistent with findings from Ventura-Comes et al., (2018) [88] who reported that elite Spanish squash players consumed ergogenic aids which had a lower efficacy such as glutamine, branch chain amino acids and flaxseed oil, rather than ones which had higher efficacy such as beta-alanine, creatine and sodium bicarbonate [89]. Players in the present study were unable to identify the rationale for use of beta-alanine supplementation (see supplementary Table 77). Beta-Alanine could enhance squash performance by increasing muscle carnosine stores and subsequent buffering capacity of the muscle [90]. Tam et al., (2019) [80] reported that supplementation was the least frequent topic of nutrition education interventions (34%). It is plausible that athletes may only take supplements recommended by their sports nutritionist, with nutritionists prioritising achieving an optimal energy intake and greater diet quality, rather than a supplementation strategy. There is also a paucity of research on the efficacy of supplements on squash-specific performance. Therefore, sports nutritionists may not recommend supplements to elite squash players due to a lack of sport-specific evidence. Future research should aim to quantify the efficacy of ergogenic aids which have been shown to augment high intensity intermittent exercise (e.g. beta-alanine, sodium bicarbonate, creatine, caffeine and nitrates) [91] in elite squash to establish a supplementation strategy specific to the sport.

### Influencing factors of nutrition knowledge

Sex was shown to have no significant differences on overall nutrition knowledge or any subsections (Figs. 3 and 4). Sex is reported to have an equivocal influence on nutrition knowledge with some studies reporting female athletes to have greater nutrition knowledge than male counterparts [26, 27, 31, 32, 37, 39, 46, 49], whereas other studies convey no differences between sexes [33–36, 38, 40–45, 47, 48]. Assuming appropriate energy availability [6], aside from iron intake in regularly menstruating females [92], the main determinants of a player’s nutritional requirements are based on their training load, regardless of sex [93]. Therefore, nutritional education requirements or nutrition knowledge shouldn’t differ between sexes in elite squash players.

World ranking was shown to have a weak positive association with nutrition knowledge (r = .208). The influence of athlete ability on nutrition knowledge is equivocal with some studies showing a greater standard of athlete to possess greater nutrition knowledge [39, 46, 50], some studies conveying no differences [28, 44, 51–54] and others reporting non-elite Australian Football athletes to have greater nutrition knowledge than elite counterparts [30]. Differences in athlete ability and nutrition knowledge may be confounded by influencing factors. Many studies quantifying the nutrition knowledge of athletes are undertaken in tertiary educated individuals (e.g. collegiate athletes), with this level of education being shown to positively influence nutrition knowledge [12]. Sub-elite and recreational individuals are also anticipated to spend less time training than elite counterparts, potentially having more time for educational purposes.

Age was also shown to have a weak positive association with nutrition knowledge (r = .281) The influence of age on nutrition knowledge is equivocal with some studies reporting older athletes to display greater nutrition knowledge [46, 55, 57] and others reporting no differences [27, 41, 44, 56]. Older athletes may have greater nutrition knowledge as time is required to be able to progress to higher levels of education (e.g. tertiary). Players may also not have access to a sports nutritionist in the early years of their career, as national governing bodies prioritise senior players who have a greater likelihood of achieving success, reducing the opportunity to increase their nutrition knowledge.

Players that had a relevant undergraduate degree reported higher nutrition knowledge than those that had no relevant qualifications (Fig. 5). This is to be expected given that undergraduate degrees (e.g. BSc Sport & Exercise Science) are designed to increase knowledge in a subject specific area, and many players cited that they had completed a nutrition module as part of their course. This is consistent with previous research which reports athletes who study or have studied at tertiary level have greater nutrition knowledge than those who have not [25–30].

Players who received their main source of nutrition information from a sport nutritionist were shown to score significantly higher than players who received their main source of nutrition information from a sport scientist, or the internet / social media (Fig. 5). This is in contrast to findings from Trakman et al., (2019) [54] who found no differences in athlete’s nutrition knowledge and main source of nutrition information. It should be noted that athletes in the aforementioned study were non-elite [54]. The level of nutrition support may vary between elite and non-elite athletes and may influence nutrition knowledge scores. Consequently, players should look to consult with a sports nutritionist to obtain nutrition information and be discouraged from using the internet / social media to increase their nutrition knowledge. Surprisingly, players who obtained their main source of nutrition information from a sports scientist were shown to have poor nutrition knowledge (44%). Sport scientists might be able to understand and communicate mechanistic underpinning. However, they may lack the ability to translate this into practical nutrition recommendations and coherent strategies for athletes.

### Limitations

A limitation of the study is that it is impossible to know whether players cheated throughout completion of the NSKQ. Players were asked to complete the NSKQ without the use of resources (peers, books, internet etc.). No member of the research team was supervising players when they completed the questionnaire due to the universal nature of the study. Therefore, it is impossible to know whether players completed NSKQ with or without the use of resources. If players were to complete the NSKQ with resources this could influence their score and provide a false result. However, the lead author would expect scored to be greater than ‘average’ if players cheated. Another limitation of the study is that the NSKQ is not specific to an individual or sport. Therefore, while players may have poor nutrition knowledge, they may have a good understanding of nutrition in relation to their sport and this is not reflected in their score. A final limitation of the study is that despite responses from a global sample of the population, the questionnaire was only distributed in English. Consequently, some players may not have been able to understand questions or may have been deterred from completing the NSKQ.

## Conclusions and future directions

This was the first study to quantify the nutrition knowledge of elite squash players. Players were found to have average nutrition knowledge (56%). Sex was shown to have no effect on players nutrition knowledge. Age and world ranking were shown to have a weak positive effect on nutrition knowledge. Players who had a relevant undergraduate degree had better nutrition knowledge than those who had no relevant education. Players who consulted with a sports nutritionist were shown to have better nutrition knowledge than those who obtained nutrition information from the internet or a sport scientist. Consequently, based on data from this study, elite squash players should aim to increase their nutrition knowledge by consulting with a sports nutritionist. Future research should aim to quantify the effectiveness of a nutrition education intervention at increasing the nutrition knowledge of elite squash players.

Players had poor knowledge of contemporary carbohydrate and protein guidelines with previous research reporting mismatches between guidelines and dietary intakes [11]. However, it is plausible that these guidelines are not specific to elite squash players and do not translate into poor dietary practices. Future research should quantify the training load and dietary practices of elite squash players to create specific nutritional recommendations for the sport. This will provide information on whether players dietary practices are optimal in relation to their training load and will create specific nutrition recommendations for elite squash players as exhibited in other racket [8] and high intensity intermittent sports [9]. This data can also be used to inform nutritional education interventions in elite squash players.

## Supplementary Information

**Additional file 1.** Individual Question Scores. RNSKQ individual question scores.

## Data Availability

Most of the data generated or analysed during this study are included in this published article [and its supplementary information files] such as overall RNSKQ scores and subsection scores as well as individual RNSKQ question scores (ST1-ST87). Individual players scores cannot be request due to identification of players.

## Abbreviations

NSKQ: Nutrition for Sport Knowledge Questionnaire
PSA: Professional Squash Association

## References

[1] Jones TW, Williams BK, Kilgallen C, Horobeanu C, Shillabeer BC, Murray A (2018). A review of the performance requirements of squash. Int J Sports Sci Coach.

[2] Lees A (2003). Science and the major racket sports: a review. J Sports Sci.

[3] Girard PO, Chevalier PR, Habrard PM, Sciberras PP, Hot PP, Millet PG (2007). Game analysis and energy requirementsl of elite squash. J Strength Cond Res.

[4] Gibson N, Bell P, Clyne A, Lobban G, Aitken L, Gibbon K (2019). Physical preparation for elite-level squash players: monitoring, assessment, and training practices for the strength and conditioning coach. Strength Cond J.

[5] James C, Dhawan A, Jones T, Pok C, Yeo V, Girard O (2021). Minimal agreement between internal and external training load metrics across a 2-wk training microcycle in elite squash. J Sports Sci Med.

[6] Mountjoy M, Sundgot-Borgen J, Burke LM, Ackerman KE, Blauwet C, Constantini N (2018). IOC consensus statement on relative energy deficiency in sport (RED-S): 2018 update. Br J Sports Med.

[7] Kerksick CM, Wilborn CD, Roberts MD, Smith-Ryan A, Kleiner SM, Jager R, et al. ISSN exercise & sports nutrition review update: research & recommendations. J Int Soc Sports Nutr. 2018;15(1):1–57. 10.1186/s12970-018-0242-y PMC6090881 30068354

[8] Ranchordas MK, Rogersion D, Ruddock A, Killer SC, Winter EM (2013). Nutrition for tennis: practical recommendations. Journal of sports science & medicine. J Sports Sci Med.

[9] Collins J, Maughan RJ, Gleeson M, Bilsborough J, Jeukendrup A, Morton JP, et al. UEFA expert group statement on nutrition in elite football. Current evidence to inform practical recommendations and guide future research. Br J Sports Med. 2020;55(8):416–22. 10.1136/bjsports-2019-101961 33097528

[10] Burke L, Hawley J, Wong S, Jeukendrup A (2011). Carbohydrates for training and competition. J Sports Sci.

[11] Ventura-Comes A, Martínez-Sanz J, Sánchez-Oliver A, Domínguez R (2019). Analysis of foods habits in squash players. J Phys Educ Sport.

[12] Trakman G, Forsyth A, Devlin B, Belski R (2016). A systematic review of athletes’ and coaches’ nutrition knowledge and reflections on the quality of current nutrition knowledge measures. Nutrients.

[13] Heaney S, O'Connor H, Michael S, Gifford J, Naughton G (2011). Nutrition knowledge in athletes: a systematic review. Int J Sport Nutr Exerc Metab.

[14] Spronk I, Heaney SE, Prvan T, O'Connor HT (2015). Relationship between general nutrition knowledge and dietary quality in elite athletes. Int J Sport Nutr Exerc Metab.

[15] Birkenhead K, Slater G (2015). A review of factors influencing athletes’ food choices. Sports Med.

[16] Basiotis PP, Welsh SO, Cronin FJ, Kelsay JL, Mertz W (1987). Number of days of food intake records required to estimate individual and group nutrient intakes with defined confidence. J Nutr.

[17] Bingham SA, Gill C, Welch A, Day K, Cassidy A, Khaw KT (1994). Comparison of dietary assessment methods in nutritional epidemiology: weighed records v. 24 h recalls, food-frequency questionnaires and estimated-diet records. Br J Nutr.

[18] Jeukendrup AE (2017). Periodized nutrition for athletes. Sports Med.

[19] Stellingwerff T, Morton JP, Burke LM (2019). A framework for Periodized nutrition for athletics. Int J Sport Nutr Exerc Metab.

[20] Heaney S, O'Connor H, Gifford J, Naughton G (2010). Comparison of strategies for assessing nutritional adequacy in elite female athletes' dietary intake. Int J Sport Nutr Exerc Metab.

[21] Valliant MW, Emplaincourt HP, Wenzel RK, Garner BH (2012). Nutrition education by a registered dietitian improves dietary intake and nutrition knowledge of a NCAA female volleyball team. Nutrients.

[22] Rossi FE, Landreth A, Beam S, Jones T, Norton L, Cholewa JM (2017). The effects of a sports nutrition education intervention on nutritional status, sport nutrition knowledge, body composition, and performance during off season training in NCAA division I baseball players. Journal of sports science & medicine. J Sports Sci Med.

[23] Trakman GL, Forsyth A, Hoye R, Belski R. The nutrition for sport knowledge questionnaire (NSKQ): development and validation using classical test theory and Rasch analysis. J Int Soc Sports Nutr. 2017;14(1):1–12. 10.1186/s12970-017-0182-y PMC5543556 28785177

[24] Trakman GL, Brown F, Forsyth A, Belski R (2019). Modifications to the nutrition for sport knowledge questionnaire (NSQK) and abridged nutrition for sport knowledge questionnaire (ANSKQ). J Int Soc Sports Nutr.

[25] Zawila LG, Steib CM, Hoogenboom B (2003). The female collegiate cross-country runner: nutritional knowledge and attitudes. Journal of athletic training. J Athl Train.

[26] Azizi M, Rahmani-Nia F, Malaee M, Malaee M, Khosravi N (2010). A study of nutritional knowledge and attitudes of elite college athletes in Iran. Braz J Biomotricity.

[27] Jessri M, Jessri M, RashidKhani B, Zinn C (2010). Evaluation of Iranian college athletes' sport nutrition knowledge. Int J Sport Nutr Exerc Metab.

[28] Andrews MC, Itsiopoulos C (2016). Room for improvement in nutrition knowledge and dietary intake of male football (soccer) players in Australia. Int J Sport Nutr Exerc Metab.

[29] Abbey EL, Wright CJ, Kirkpatrick CM (2017). Nutrition practices and knowledge among NCAA division III football players. J Int Soc Sports Nutr.

[30] Trakman G, Forsyth A, Middleton K, Hoye R, Jenner S, Keenan S (2018). Australian football athletes lack awareness of current sport nutrition guidelines. Int J Sport Nutr Exerc Metab.

[31] Douglas P, Douglas J (1984). Nutrition knowledge and food practices of high school athletes. J Am Diet Assoc.

[32] Worme J, Doubt T, Singh A, Ryan C, Moses F, Duester P (1990). Dietary patterns, gastrointestinal complaints, and nutrition knowledge of recreational triathletes. Am J Clin Nutr.

[33] Hamilton G, Thompson C, Hopkins W (1994). Nutrition knowledge of elite distance runners. NZ J Sports Med.

[34] Rosenbloom C, Jonnalagadda S, Skinner R (2002). Nutrition knowledge of collegiate athletes in a division I National Collegiate Athletic Association Institution. J Am Diet Assoc.

[35] Nichols PE, Jonnalagadda SS, Rosenbloom CA, Trinkaus M (2005). Knowledge, attitudes, and behaviors regarding hydration and fluid replacement of collegiate athletes. Int J Sport Nutr Exerc Metab.

[36] Condon E, Dube K, Herbold N (2007). The influence of low-carbohydrate trend on athletes’ knowledge, attitudes, and dietary intake of carbohydrates. Top Clin Nutr.

[37] Dunn D, Turner LW, Denny G. Nutrition knowledge and attitudes of college athletes. Sport J. 2007;10(4):1–8.

[38] Rash C, Malinauskas B, Duffrin M, Barber-Heidal K, Overton R (2008). Nutrition-related knowledge, attitude, and dietary intake of college track athletes. Sport J.

[39] Spendlove JK, Heaney SE, Gifford JA, Prvan T, Denyer GS, O'Connor HT (2012). Evaluation of general nutrition knowledge in elite Australian athletes. Br J Nutr.

[40] Sedek R, Yih T (2014). Dietary habits and nutrition knowledge among athletes and non-athletes in national university of Malaysia (UKM). Pak J Nutr.

[41] Webb MC, Beckford SE (2014). Nutritional knowledge and attitudes of adolescent swimmers in Trinidad and Tobago. Journal of nutrition and metabolism. J Nutr Metab.

[42] Walker N, Love TD, Baker DF, Healey PB, Haszard J, Edwards AS (2014). Knowledge and attitudes to vitamin D and sun exposure in elite New Zealand athletes: a cross-sectional study. J Int Soc Sports Nutr.

[43] Weeden A, Olsen J, Batacan J, Peterson T (2014). Differences in collegiate athlete nutrition knowledge as determined by athlete characteristics. Sport J.

[44] Hardy R, Kliemann N, Evansen T, Brand J (2016). Relationship between energy drink consumption and nutrition knowledge in student-athletes. J Nutr Educ Behav.

[45] Manore MM, Patton-Lopez M, Meng Y, Wong SS (2017). Sport nutrition knowledge, behaviors and beliefs of high school soccer players. Nutrients.

[46] Heikkilä M, Valve R, Lehtovirta M, Fogelholm M (2018). Nutrition knowledge among young Finnish endurance athletes and their coaches. Int J Sport Nutr Exerc Metab.

[47] Patton-Lopez M, Manore MM, Branscum A, Meng Y, Wong SS (2018). Changes in sport nutrition knowledge, attitudes/beliefs and behaviors following a two-year sport nutrition education and life-skills intervention among high school soccer players. Nutrients.

[48] Blennerhassett C, McNaughton LR, Cronin L, Sparks SA (2018). Development and implementation of a nutrition knowledge questionnaire for Ultraendurance athletes. Int J Sport Nutr Exerc Metab.

[49] Citarella R, Itani L, Intini V, Zucchinali G, Scevaroli S, Kreidieh D (2019). Nutritional knowledge and dietary practice in elite 24-hour Ultramarathon runners: a brief report. Sports (Basel).

[50] Harrison J, Hopkins W, MacFarlane D, Worsley A (1991). Nutrition knowledge and dietary habits of elite and non-elite athletes. Aust J Nutr Diet.

[51] Hoogenboom BJ, Morris J, Morris C, Schaefer K (2009). Nutritional knowledge and eating behaviors of female, collegiate swimmers. North American journal of sports physical therapy. N Am J Sports Phys Ther.

[52] Devlin BL, Leveritt MD, Kingsley M, Belski R (2017). Dietary intake, body composition, and nutrition knowledge of Australian football and soccer players: implications for sports nutrition professionals in practice. Int J Sport Nutr Exerc Metab.

[53] Lohman R, Carr A, Condo D (2019). Nutritional intake in Australian football players: sports nutrition knowledge and macronutrient and micronutrient intake. Int J Sport Nutr Exerc Metab.

[54] Trakman GL, Forsyth A, Hoye R, Belski R (2019). Australian team sports athletes prefer dietitians, the internet and nutritionists for sports nutrition information. Nutrition & dietetics. Nutr Diet.

[55] Reading KJ, McCargar LJ, Marriage BJ (1999). Adolescent and young adult male hockey players: nutrition knowledge and education. Canadian journal of dietetic practice and research. Can J Diet Pract Res.

[56] Devlin BL, Belski R (2015). Exploring general and sports nutrition and food knowledge in elite male Australian athletes. Int J Sport Nutr Exerc Metab.

[57] Argôlo D, Borges J, Cavalcante A, Silva G, Maia S, Moraes A (2018). Poor dietary intake and low nutritional knowledge in adolescent and adult competitive athletes: a warning to table tennis players. Nutr Hosp.

[58] World Medical Association (2001). Declaration of Helsinki: ethical principles for medical research involving human subjects. Bull World Health Organ.

[59] Wu M, Tam HP, Jen TH (2017). Chapter 5, classical test theory. Educational measurement for applied researchers.

[60] Boone WJ, Bolan E (2016). Rasch analysis for instrument development: why, when, and how?. CBE Life Sci Educ.

[61] Doering TM, Reaburn PR, Cox G, Jenkins DG (2016). Comparison of Postexercise nutrition knowledge and Postexercise carbohydrate and protein intake between Australian masters and younger triathletes. Int J Sport Nutr Exerc Metab.

[62] Eskici G, Ersoy G (2016). An evaluation of wheelchair basketball players’ nutritional status and nutritional knowledge levels. J Sports Med Phys Fitness.

[63] Folasire OF, Akomolafe AA, Sanusi RA (2015). Does nutrition knowledge and practice of athletes translate to enhanced athletic performance? Cross-sectional study amongst Nigerian undergraduate athletes. Global journal of health science. Global J Health Sci.

[64] Jacobson BH, Sobonya C, Ransone J (2001). Nutrition practices and knowledge of college varsity athletes: a follow-up. Journal of strength and conditioning research. J Strength Cond Res.

[65] Jenner SL, Trakman G, Coutts A, Kempton T, Ryan S, Forsyth A, et al. Dietary intake of professional Australian football athletes surrounding body composition assessment. J Int Soc Sports Nutr. 2018;15(1):1–8. 10.1186/s12970-018-0248-5 PMC6137941 30217203

[66] Judge LW, Kumley RF, Bellar DM, Pike KL, Pierson EE, Weidner T (2016). Hydration and fluid replacement knowledge, attitudes, barriers, and behaviors of NCAA division 1 American football players. Journal of strength and conditioning research. J Strength Cond Res.

[67] Kelly VG, Leveritt MD, Brennan CT, Slater GJ, Jenkins DG (2016). Prevalence, knowledge and attitudes relating to β -alanine use among professional footballers. J Sci Med Sport.

[68] McCubbin AJ, Cox GR, Costa RJS (2019). Sodium intake beliefs, information sources, and intended practices of endurance athletes before and during exercise. Int J Sport Nutr Exerc Metab.

[69] Cohen J (1988). Statistical power analysis for the behavioral sciences.

[70] Schober P, Boer C, Lothar A (2018). Correlation coefficients: appropriate use and interpretation. Anesth Analg.

[71] Chapman P, Toma RB, Tuveson RV, Jacob M (1997). Nutrition knowledge among adolescent high school female athletes. Adolescence.

[72] Doyle-Lucas A, Davy BM (2011). Development and evaluation of an educational intervention program for pre-professional adolescent ballet dancers: nutrition for optimal performance. Journal of dance medicine & science. J Dance Med Sci.

[73] Daniel NVS, Jürgensen LP, Padovani RDC, Juzwiak CR (2016). Impact of an interdisciplinary food, nutrition and health education program for adolescent Brazilian volleyball players. Rev Nutr.

[74] Nascimento M, Silva D, Ribeiro S, Nunes M, Almeida M, Mendes-Netto R (2016). Effect of a nutritional intervention in Athlete's body composition, eating behaviour and nutritional knowledge: a comparison between adults and adolescents. Nutrients.

[75] Philippou E, Middleton N, Pistos C, Andreou E, Petrou M (2017). The impact of nutrition education on nutrition knowledge and adherence to the mediterranean diet in adolescent competitive swimmers. J Sci Med Sport.

[76] Heikkilä M, Lehtovirta M, Autio O, Fogelholm M, Valve R (2019). The impact of nutrition education intervention with and without a Mobile phone application on nutrition knowledge among young endurance athletes. Nutrients.

[77] Kunkel ME, Bell LB, Luccia BHD (2001). Peer nutrition education program to improve nutrition knowledge of female collegiate athletes. J Nutr Educ.

[78] Abood DA, Black DR, Birnbaum RD (2004). Nutrition education intervention for college female athletes. Journal of nutrition education and behavior. J Nutr Educ Behav.

[79] Simpson A, Gemming L, Baker D, Braakhuis A (2017). Do image-assisted Mobile applications improve dietary habits, knowledge, and Behaviours in elite athletes? A pilot study. Sports (Basel).

[80] Tam R, Beck K, Manore M, Gifford J, Flood V, O’Connor H (2019). Effectiveness of education interventions designed to improve nutrition knowledge in athletes: a systematic review. Sports Med.

[81] McCrink CM, McSorley EM, Grant K, McNeilly AM, Magee PJ. An investigation of dietary intake, nutrition knowledge and hydration status of Gaelic football players. Eur J Nutr. 2020;60(3):1465–73. 10.1007/s00394-020-02341-x PMC7987599 32734346

[82] Phillips SM (2012). Dietary protein requirements and adaptive advantages in athletes. Br J Nutr.

[83] Spronk I, Kullen C, Burdon C, O'Connor H (2014). Relationship between nutrition knowledge and dietary intake. Br J Nutr.

[84] Morehen JC, Bradley WJ, Clarke J, Twist C, Hambly C, Speakman JR (2016). The assessment of Total energy expenditure during a 14-Day in-season period of professional Rugby league players using the doubly labelled water method. Int J Sport Nutr Exerc Metab.

[85] Anderson L, Orme P, Naughton RJ, Close GL, Milsom J, Rydings D (2017). Energy intake and expenditure of professional soccer players of the English premier league: evidence of carbohydrate periodization. Int J Sport Nutr Exerc Metab.

[86] Thompson FE, Subar AF, Coulston AM (2017). Dietary assessment methodology. Nutrition in the prevention and treatment of disease.

[87] Costello N, Deighton K, Dyson J, Mckenna J, Jones B (2017). Snap-N-send: a valid and reliable method for assessing the energy intake of elite adolescent athletes. Eur J Sport Sci.

[88] Ventura-Comes A, Martínez-Sanz J, Sánchez-Oliver A, Domínguez R (2018). Analysis of nutritional supplements consumption by squash players. Nutrients.

[89] Maughan RJ, Burke LM, Dvorak J, Larson-Meyer D, Peeling P, Phillips SM (2018). IOC consensus statement: dietary supplements and the high-performance athlete. Br J Sports Med.

[90] Saunders B, Elliott-Sale K, Artioli GG, Swinton PA, Dolan E, Roschel H (2017). β-Alanine supplementation to improve exercise capacity and performance: a systematic review and meta-analysis. Br J Sports Med.

[91] Forbes SC, Candow DG, Smith-Ryan A, Hirsch KR, Roberts MD, VanDusseldorp TA (2020). Supplements and nutritional interventions to augment high-intensity interval training physiological and performance adaptations-a narrative review. Nutrients.

[92] Sim M, Garvican-Lewis L, Cox GR, Govus A, McKay AKA, Stellingwerff T (2019). Iron considerations for the athlete: a narrative review. Eur J Appl Physiol.

[93] Desbrow B, Burd NA, Tarnopolsky M, Moore DR, Elliott-Sale K (2019). Nutrition for special populations: young, female, and masters athletes. Int J Sport Nutr Exerc Metab.

