# Supplementary material for: Elite squash players nutrition knowledge and influencing factors
Source: J Int Soc Sports Nutr. 2021 Jun 10;18:46. doi: 10.1186/s12970-021-00443-3 (PMC8194110; doi:10.1186/s12970-021-00443-3)
Supplement: Supplementary file 1 — Additional file 1. Individual Question Scores. RNSKQ individual question scores. [file 12970_2021_443_MOESM1_ESM.docx]

**INDIVIDUAL QUESTION SCORES**

*Supplementary Table 1. Players Answers to Q1.1 Which nutrient do you think has the most energy (kilojoules/calories) per 100 grams (3.5 ounces)?*

| **Carbohydrate** | | **Protein** | | **Fat*** | | **Not sure** | |
| --- | --- | --- | --- | --- | --- | --- | --- |
| ***n*** | **%** | ***n*** | **%** | ***n*** | **%** | ***n*** | **%** |
| 45 | 58.44 | 5 | 6.49 | 26 | 33.77 | 1 | 1.30 |

*Denotes correct answer

*Supplementary Table 2. Players Answers to Q1.2 Do you agree or disagree with the following statements about weight loss? Having the lowest weight possible benefits endurance performance in the long term*

| **Agree** | | **Disagree*** | | **Not sure** | |
| --- | --- | --- | --- | --- | --- |
| ***n*** | **%** | ***n*** | **%** | ***n*** | **%** |
| 13 | 16.88 | 59 | 76.62 | 5 | 6.49 |

*Denotes correct answer

*Supplementary Table 3. Players Answers to Q1.3 Do you agree or disagree with the following statements about weight loss? Eating more protein is the most important dietary change if you want to have more muscle*

| **Agree** | | **Disagree*** | | **Not sure** | |
| --- | --- | --- | --- | --- | --- |
| ***n*** | **%** | ***n*** | **%** | ***n*** | **%** |
| 60 | 77.92 | 15 | 19.48 | 2 | 2.60 |

*Denotes correct answer

*Supplementary Table 4. Players Answers to Q1.4 Do you agree or disagree with the following statements about weight loss? Eating more energy from protein than you need can make you put on fat*

| **Agree*** | | **Disagree** | | **Not sure** | |
| --- | --- | --- | --- | --- | --- |
| ***n*** | **%** | ***n*** | **%** | ***n*** | **%** |
| 39 | 50.65 | 24 | 31.17 | 14 | 18.18 |

*Denotes correct answer

*Supplementary Table 5. Players Answers to Q1.5 Do you think the diet changes below are good ways to lose weight? Swapping carbohydrates/energy dense foods for low-energy foods like vegetables*

| **Yes*** | | **No** | | **Not sure** | |
| --- | --- | --- | --- | --- | --- |
| ***n*** | **%** | ***n*** | **%** | ***n*** | **%** |
| 48 | 62.34 | 27 | 35.06 | 2 | 2.60 |

*Denotes correct answer

*Supplementary Table 6. Players Answers to Q1.6 Do you think the diet changes below are good ways to lose weight? Eating margarine instead of butter*

| **Yes** | | **No*** | | **Not sure** | |
| --- | --- | --- | --- | --- | --- |
| ***n*** | **%** | ***n*** | **%** | ***n*** | **%** |
| 23 | 29.87 | 36 | 46.75 | 23 | 29.87 |

*Denotes correct answer

*Supplementary Table 7. Players Answers to Q1.7 Do you think the diet changes below are good ways to lose weight? Eating protein bars and shakes instead of yogurts, muesli/granola bars and fruits*

| **Yes** | | **No*** | | **Not sure** | |
| --- | --- | --- | --- | --- | --- |
| ***n*** | **%** | ***n*** | **%** | ***n*** | **%** |
| 7 | 9.09 | 69 | 89.61 | 1 | 1.30 |

*Denotes correct answer

*Supplementary Table 8. Players Answers to Q1.8 Do you think the diet changes below are good ways to lose weight? Choosing lower glycemic index (GI) carbohydrates to help regulate appetite*

| **Yes*** | | **No** | | **Not sure** | |
| --- | --- | --- | --- | --- | --- |
| ***n*** | **%** | ***n*** | **%** | ***n*** | **%** |
| 47 | 61.04 | 8 | 10.39 | 22 | 28.57 |

*Denotes correct answer

*Supplementary Table 9. Players Answers to Q1.9 If they want to lose weight, athletes should:*

| **Eat less than 50 grams (1.7 ounces) of carbohydrate per day** | | **Eat less than 20 g (0.7 ounces) of fat per day** | | **Eat less calories / kilojoules than your body needs*** | | **Not sure** | |
| --- | --- | --- | --- | --- | --- | --- | --- |
| ***n*** | **%** | ***n*** | **%** | ***n*** | **%** | ***n*** | **%** |
| 6 | 7.79 | 12 | 15.58 | 45 | 58.44 | 14 | 18.18 |

*Denotes correct answer

*Supplementary Table 10. Players Answers to Q1.10 To ensure they meet their energy (kilojoule/calorie) requirements, all athletes should:*

| **Plan their diet based on their age, gender, body size, sport and training program*** | | **Eat based on their hunger and fulness signals** | | **Not sure** | |
| --- | --- | --- | --- | --- | --- |
| ***n*** | **%** | ***n*** | **%** | ***n*** | **%** |
| 75 | 97.40 | 2 | 2.60 | 0 | 0 |

*Denotes correct answer

*Supplementary Table 11. Players Answers to Q1.11 Which is a better recovery meal option for an athlete who wants to put on muscle?*

| **A ‘mass gainer’ shake and 3-4 scrambled eggs** | | **Pasta with lean beef and vegetable sauce, plus a dessert of fruit, yogurt and nuts*** | | **A large piece of grilled chicken with side salad (lettuce, cucumber, tomato)** | | **A large steak and fried eggs** | | **Not sure** | |
| --- | --- | --- | --- | --- | --- | --- | --- | --- | --- |
| ***n*** | **%** | ***n*** | **%** | ***n*** | **%** | ***n*** | ***%*** | ***n*** | **%** |
| 17 | 22.08 | 34 | 44.16 | 16 | 20.78 | 6 | 7.79 | 4 | 5.19 |

*Denotes correct answer

*Supplementary Table 12. Players Answers to Q1.12 Which is a better recovery meal option for an athlete who wants to lose weight?*

| **A side salad with no dressing (lettuce, cucumber, tomato)** | | **A pure whey protein isolate (WPI) shake made with water** | | **A mixed meal that includes a small-moderate serving of meat and carbohydrate plus a large side salad*** | | **Not sure** | |
| --- | --- | --- | --- | --- | --- | --- | --- |
| ***n*** | **%** | ***n*** | **%** | ***n*** | **%** | ***n*** | **%** |
| 3 | 3.90 | 10 | 12.99 | 62 | 80.50 | 2 | 2.60 |

*Denotes correct answer

*Supplementary Table 13. Players Answers to Q2.1 An athlete doing a moderate to high-intensity endurance training program for about two hours should eat…*

| **1-3 g carbohydrate per kg (0.016-0.048 ounces per lb) bodyweight per day** | | **5-7 g/kg, increasing up to 10 g/kg with intense training competition loads of carbohydrate per day*** | | **75-85 % of total daily kilojoule / calorie intake as carbohydrate** | | **Not sure** | |
| --- | --- | --- | --- | --- | --- | --- | --- |
| ***n*** | **%** | ***n*** | **%** | ***n*** | **%** | ***n*** | **%** |
| 17 | 22.08 | 34 | 44.16 | 4 | 5.19 | 22 | 28.57 |

*Denotes correct answer

*Supplementary Table 14. Players Answers to Q2.2 Which options have enough carbohydrate for recovery from about 1 hour of high intensity aerobic exercise? Assume the athlete weighs about 70kg and has an important training session again tomorrow; 1 medium banana?*

| **Enough** | | **Not enough*** | | **Not sure** | |
| --- | --- | --- | --- | --- | --- |
| ***n*** | **%** | ***n*** | **%** | ***n*** | **%** |
| 13 | 16.88 | 60 | 77.92 | 4 | 5.19 |

*Denotes correct answer

*Supplementary Table 15. Players Answers to Q2.3 Which options have enough carbohydrate for recovery from about 1 hour of high intensity aerobic exercise? Assume the athlete weighs about 70kg and has an important training session again tomorrow; 1 cup of quinoa and 1 tin of tuna?*

| **Enough** | | **Not enough*** | | **Not sure** | |
| --- | --- | --- | --- | --- | --- |
| ***n*** | **%** | ***n*** | **%** | ***n*** | **%** |
| 60 | 77.92 | 12 | 15.58 | 5 | 6.49 |

*Denotes correct answer

*Supplementary Table 16. Players Answers to Q2.4 Which options have enough carbohydrate for recovery from about 1 hour of high intensity aerobic exercise? Assume the athlete weighs about 70kg and has an important training session again tomorrow; 1 cup of plain yogurt?*

| **Enough** | | **Not enough*** | | **Not sure** | |
| --- | --- | --- | --- | --- | --- |
| ***n*** | **%** | ***n*** | **%** | ***n*** | **%** |
| 3 | 3.90 | 71 | 92.21 | 3 | 3.90 |

*Denotes correct answer

*Supplementary Table 17. Players Answers to Q2.5 Which options have enough carbohydrate for recovery from about 1 hour of high intensity aerobic exercise? Assume the athlete weighs about 70kg and has an important training session again tomorrow; 1 cup of baked beans on two slices of bread?*

| **Enough*** | | **Not enough** | | **Not sure** | |
| --- | --- | --- | --- | --- | --- |
| ***n*** | **%** | ***n*** | **%** | ***n*** | **%** |
| 57 | 74.03 | 13 | 16.88 | 7 | 9.09 |

*Denotes correct answer

*Supplementary Table 18. Players Answers to Q2.6 Which food has the most carbohydrate?*

| **One cup (168 g / 5.6 ounces) boiled rice*** | | **Two slices of white sandwich loaf bread** | | **One medium (150 g / 5 ounces) boiled potato** | | **1 medium (150 g / 5 ounces) ripe banana** | | **Not sure** | |
| --- | --- | --- | --- | --- | --- | --- | --- | --- | --- |
| ***n*** | **%** | ***n*** | **%** | ***n*** | **%** | ***n*** | ***%*** | ***n*** | **%** |
| 33 | 42.86 | 15 | 19.48 | 16 | 20.78 | 6 | 7.79 | 7 | 9.09 |

*Denotes correct answer

*Supplementary Table 19. Players Answers to Q2.7 Do you agree or disagree with these statements about fat; The body needs fat to fight off sickness?*

| **Agree*** | | **Disagree** | | **Not sure** | |
| --- | --- | --- | --- | --- | --- |
| ***n*** | **%** | ***n*** | **%** | ***n*** | **%** |
| 36 | 46.75 | 18 | 23.38 | 23 | 29.87 |

*Denotes correct answer

*Supplementary Table 20. Players Answers to Q2.8 Do you agree or disagree with these statements about fat; Athletes should not eat more than 20g of fat per day*

| **Agree** | | **Disagree*** | | **Not sure** | |
| --- | --- | --- | --- | --- | --- |
| ***n*** | **%** | ***n*** | **%** | ***n*** | **%** |
| 15 | 19.48 | 41 | 53.25 | 21 | 27.27 |

*Denotes correct answer

*Supplementary Table 21. Players Answers to Q2.9 Do you agree or disagree with these statements about fat; When we increase the intensity of exercise, the % of fat we use as a fuel also increases*

| **Agree** | | **Disagree*** | | **Not sure** | |
| --- | --- | --- | --- | --- | --- |
| ***n*** | **%** | ***n*** | **%** | ***n*** | **%** |
| 40 | 51.95 | 34 | 44.16 | 3 | 3.90 |

*Denotes correct answer

*Supplementary Table 22. Players Answers to Q2.10 Do you agree or disagree with these statements about fat; When we exercise at a low intensity, our body mostly uses fat as a fuel*

| **Agree*** | | **Disagree** | | **Not sure** | |
| --- | --- | --- | --- | --- | --- |
| ***n*** | **%** | ***n*** | **%** | ***n*** | **%** |
| 42 | 54.55 | 26 | 33.77 | 9 | 11.69 |

*Denotes correct answer

*Supplementary Table 23. Players Answers to Q2.11 Do you think these foods are high in fat; Cheddar cheese?*

| **Yes*** | | **No** | | **Not sure** | |
| --- | --- | --- | --- | --- | --- |
| ***n*** | **%** | ***n*** | **%** | ***n*** | **%** |
| 72 | 93.51 | 3 | 3.90 | 2 | 2.60 |

*Denotes correct answer

*Supplementary Table 24. Players Answers to Q2.12 Do you think these foods are high in fat; Margarine?*

| **Yes*** | | **No** | | **Not sure** | |
| --- | --- | --- | --- | --- | --- |
| ***n*** | **%** | ***n*** | **%** | ***n*** | **%** |
| 53 | 68.83 | 17 | 22.08 | 7 | 9.09 |

*Denotes correct answer

*Supplementary Table 25. Players Answers to Q2.13 Do you think these foods are high in fat; Mixed nuts?*

| **Yes*** | | **No** | | **Not sure** | |
| --- | --- | --- | --- | --- | --- |
| ***n*** | **%** | ***n*** | **%** | ***n*** | **%** |
| 63 | 81.82 | 12 | 15.58 | 2 | 2.60 |

*Denotes correct answer

*Supplementary Table 26. Players Answers to Q2.14 Do you think these foods are high in fat; Honey?*

| **Yes** | | **No*** | | **Not sure** | |
| --- | --- | --- | --- | --- | --- |
| ***n*** | **%** | ***n*** | **%** | ***n*** | **%** |
| 9 | 11.69 | 59 | 76.62 | 9 | 11.69 |

*Denotes correct answer

*Supplementary Table 27. Players Answers to Q2.15 Do you agree or disagree with the statements about protein? Protein is the main fuel that muscles use during exercise*

| **Agree** | | **Disagree*** | | **Not sure** | |
| --- | --- | --- | --- | --- | --- |
| ***n*** | **%** | ***n*** | **%** | ***n*** | **%** |
| 20 | 25.97 | 53 | 68.83 | 4 | 5.19 |

*Denotes correct answer

*Supplementary Table 28. Players Answers to Q2.16 Do you agree or disagree with the statements about protein? Vegetarian athletes can meet their protein requirements without the use of protein supplements*

| **Agree*** | | **Disagree** | | **Not sure** | |
| --- | --- | --- | --- | --- | --- |
| ***n*** | **%** | ***n*** | **%** | ***n*** | **%** |
| 64 | 83.12 | 10 | 12.99 | 3 | 3.90 |

*Denotes correct answer

*Supplementary Table 29. Players Answers to Q2.17 Do you agree or disagree with the statements about protein? An experienced athlete needs more protein than a young athlete who is just starting training*

| **Agree** | | **Disagree*** | | **Not sure** | |
| --- | --- | --- | --- | --- | --- |
| ***n*** | **%** | ***n*** | **%** | ***n*** | **%** |
| 23 | 29.87 | 43 | 55.84 | 11 | 14.29 |

*Denotes correct answer

*Supplementary Table 30. Players Answers to Q2.18 Do you agree or disagree with the statements about protein? The body has a limited ability to use protein for muscle protein synthesis*

| **Agree*** | | **Disagree** | | **Not sure** | |
| --- | --- | --- | --- | --- | --- |
| ***n*** | **%** | ***n*** | **%** | ***n*** | **%** |
| 48 | 62.34 | 8 | 10.39 | 21 | 27.27 |

*Denotes correct answer

*Supplementary Table 31. Players Answers to Q2.19 Do you agree or disagree with the statements about protein? A balanced diet with enough kilojoules/calories (energy) has enough protein for most athletes*

| **Agree*** | | **Disagree** | | **Not sure** | |
| --- | --- | --- | --- | --- | --- |
| ***n*** | **%** | ***n*** | **%** | ***n*** | **%** |
| 43 | 62.34 | 24 | 31.17 | 10 | 12.99 |

*Denotes correct answer

*Supplementary Table 32. Players Answers to Q2.20 Which food has the most protein?*

| **Two eggs** | | **100 g (3 ounces) of raw skinless chicken breast*** | | **30 g (1 ounce) of almonds** | | **Not sure** | |
| --- | --- | --- | --- | --- | --- | --- | --- |
| ***n*** | **%** | ***n*** | **%** | ***n*** | **%** | ***n*** | **%** |
| 14 | 18.18 | 51 | 66.23 | 5 | 6.49 | 7 | 9.09 |

*Denotes correct answer

*Supplementary Table 33. Players Answers to Q2.21 The protein needs of a 100 kg (220 lb) well trained resistance athlete are closest to:*

| **100 g (1 g/kg)** | | **150 g (1.5 g/kg)*** | | **500 g (5 g/kg)** | | **They should eat as much protein as possible** | | **Not sure** | |
| --- | --- | --- | --- | --- | --- | --- | --- | --- | --- |
| ***n*** | **%** | ***n*** | **%** | ***n*** | **%** | ***n*** | ***%*** | ***n*** | **%** |
| 8 | 10.39 | 38 | 49.35 | 11 | 14.29 | 4 | 5.19 | 16 | 20.78 |

*Denotes correct answer

*Supplementary Table 34. Players Answers to Q2.22 Which of these foods do you think have enough protein to promote muscle growth after a bout of resistance exercise? 100g (3 ounces) chicken breast*

| **Enough*** | | **Not enough** | | **Not sure** | |
| --- | --- | --- | --- | --- | --- |
| ***n*** | **%** | ***n*** | **%** | ***n*** | **%** |
| 72 | 93.51 | 5 | 6.49 | 0 | 0 |

*Denotes correct answer

*Supplementary Table 35. Players Answers to Q2.23 Which of these foods do you think have enough protein to promote muscle growth after a bout of resistance exercise? 30g (1 ounce) Yellow cheese*

| **Enough** | | **Not enough*** | | **Not sure** | |
| --- | --- | --- | --- | --- | --- |
| ***n*** | **%** | ***n*** | **%** | ***n*** | **%** |
| 11 | 14.29 | 56 | 72.73 | 10 | 12.99 |

*Denotes correct answer

*Supplementary Table 36. Players Answers to Q2.24 Which of these foods do you think have enough protein to promote muscle growth after a bout of resistance exercise? 1 cup baked beans*

| **Enough** | | **Not enough*** | | **Not sure** | |
| --- | --- | --- | --- | --- | --- |
| ***n*** | **%** | ***n*** | **%** | ***n*** | **%** |
| 38 | 49.35 | 32 | 41.56 | 7 | 9.09 |

*Denotes correct answer

*Supplementary Table 37. Players Answers to Q2.25 Which of these foods do you think have enough protein to promote muscle growth after a bout of resistance exercise? 1/2 cup cooked quinoa*

| **Enough** | | **Not enough*** | | **Not sure** | |
| --- | --- | --- | --- | --- | --- |
| ***n*** | **%** | ***n*** | **%** | ***n*** | **%** |
| 19 | 24.68 | 53 | 68.83 | 5 | 6.49 |

*Denotes correct answer

*Supplementary Table 38. Players Answers to Q2.26 Do you think these foods have all the essential amino acids needed by the body? Beef steak*

| **Yes*** | | **No** | | **Not sure** | |
| --- | --- | --- | --- | --- | --- |
| ***n*** | **%** | ***n*** | **%** | ***n*** | **%** |
| 40 | 51.95 | 25 | 32.47 | 12 | 15.58 |

*Denotes correct answer

*Supplementary Table 39. Players Answers to Q2.27 Do you think these foods have all the essential amino acids needed by the body? Eggs*

| **Yes*** | | **No** | | **Not sure** | |
| --- | --- | --- | --- | --- | --- |
| ***n*** | **%** | ***n*** | **%** | ***n*** | **%** |
| 44 | 57.14 | 20 | 25.97 | 13 | 16.88 |

*Denotes correct answer

*Supplementary Table 40. Players Answers to Q2.28 Do you think these foods have all the essential amino acids needed by the body? Lentils*

| **Yes** | | **No*** | | **Not sure** | |
| --- | --- | --- | --- | --- | --- |
| ***n*** | **%** | ***n*** | **%** | ***n*** | **%** |
| 33 | 42.86 | 32 | 41.56 | 12 | 15.58 |

*Denotes correct answer

*Supplementary Table 41. Players Answers to Q2.29 Do you think these foods have all the essential amino acids needed by the body? Cow’s milk*

| **Yes*** | | **No** | | **Not sure** | |
| --- | --- | --- | --- | --- | --- |
| ***n*** | **%** | ***n*** | **%** | ***n*** | **%** |
| 37 | 48.05 | 27 | 35.06 | 13 | 16.88 |

*Denotes correct answer

*Supplementary Table 42. Players Answers to Q2.30 The amount of protein in skim milk compared to full cream milk is:*

| **Much less** | | **About the same*** | | **Much more** | | **Not sure** | |
| --- | --- | --- | --- | --- | --- | --- | --- |
| ***n*** | **%** | ***n*** | **%** | ***n*** | **%** | ***n*** | **%** |
| 12 | 15.58 | 39 | 50.65 | 9 | 11.69 | 17 | 22.08 |

*Denotes correct answer

*Supplementary Table 43. Players Answers to Q3.1 Do you agree or disagree with these statements on vitamins and minerals? Calcium is the main component of bone*

| **Agree*** | | **Disagree** | | **Not sure** | |
| --- | --- | --- | --- | --- | --- |
| ***n*** | **%** | ***n*** | **%** | ***n*** | **%** |
| 64 | 83.12 | 6 | 7.79 | 7 | 9.09 |

*Denotes correct answer

*Supplementary Table 44. Players Answers to Q3.2 Do you agree or disagree with these statements on vitamins and minerals? Vitamin C is an antioxidant*

| **Agree*** | | **Disagree** | | **Not sure** | |
| --- | --- | --- | --- | --- | --- |
| ***n*** | **%** | ***n*** | **%** | ***n*** | **%** |
| 47 | 61.04 | 11 | 14.29 | 19 | 24.68 |

*Denotes correct answer

*Supplementary Table 45. Players Answers to Q3.3 Do you agree or disagree with these statements on vitamins and minerals? Thiamine (Vitamin B1) is needed to take oxygen to muscles*

| **Agree** | | **Disagree*** | | **Not sure** | |
| --- | --- | --- | --- | --- | --- |
| ***n*** | **%** | ***n*** | **%** | ***n*** | **%** |
| 22 | 28.57 | 14 | 18.18 | 41 | 53.25 |

*Denotes correct answer

*Supplementary Table 46. Players Answers to Q3.4 Do you agree or disagree with these statements on vitamins and minerals? Iron is needed to turn food into usable energy*

| **Agree** | | **Disagree*** | | **Not sure** | |
| --- | --- | --- | --- | --- | --- |
| ***n*** | **%** | ***n*** | **%** | ***n*** | **%** |
| 27 | 35.06 | 24 | 31.17 | 26 | 33.77 |

*Denotes correct answer

*Supplementary Table 47. Players Answers to Q3.5 Do you agree or disagree with these statements on vitamins and minerals? Vitamin D enhances calcium absorption*

| **Agree*** | | **Disagree** | | **Not sure** | |
| --- | --- | --- | --- | --- | --- |
| ***n*** | **%** | ***n*** | **%** | ***n*** | **%** |
| 43 | 55.84 | 7 | 9.09 | 27 | 35.06 |

*Denotes correct answer

*Supplementary Table 48. Players Answers to Q3.6 Do you agree or disagree with these statements on vitamins and minerals? Meat, chicken and fish are good sources of zinc*

| **Agree*** | | **Disagree** | | **Not sure** | |
| --- | --- | --- | --- | --- | --- |
| ***n*** | **%** | ***n*** | **%** | ***n*** | **%** |
| 49 | 63.64 | 7 | 9.09 | 21 | 27.27 |

*Denotes correct answer

*Supplementary Table 49. Players Answers to Q3.7 Do you agree or disagree with these statements on vitamins and minerals? Wholegrain foods are good sources of vitamin C*

| **Agree** | | **Disagree*** | | **Not sure** | |
| --- | --- | --- | --- | --- | --- |
| ***n*** | **%** | ***n*** | **%** | ***n*** | **%** |
| 18 | 23.38 | 36 | 46.75 | 23 | 29.87 |

*Denotes correct answer

*Supplementary Table 50. Players Answers to Q3.8 Do you agree or disagree with these statements on vitamins and minerals? Fruit and vegetables are good sources of calcium*

| **Agree** | | **Disagree*** | | **Not sure** | |
| --- | --- | --- | --- | --- | --- |
| ***n*** | **%** | ***n*** | **%** | ***n*** | **%** |
| 16 | 20.78 | 49 | 63.64 | 12 | 15.58 |

*Denotes correct answer

*Supplementary Table 51. Players Answers to Q3.9 Do you agree or disagree with these statements on vitamins and minerals? Fatty fish is a good source of vitamin D*

| **Agree*** | | **Disagree** | | **Not sure** | |
| --- | --- | --- | --- | --- | --- |
| ***n*** | **%** | ***n*** | **%** | ***n*** | **%** |
| 40 | 51.95 | 21 | 27.27 | 16 | 20.78 |

*Denotes correct answer

*Supplementary Table 52. Players Answers to Q3.10 Do you agree or disagree with these statements on vitamins and minerals? Women who have a monthly period need more iron than men*

| **Agree*** | | **Disagree** | | **Not sure** | |
| --- | --- | --- | --- | --- | --- |
| ***n*** | **%** | ***n*** | **%** | ***n*** | **%** |
| 56 | 72.73 | 4 | 5.19 | 17 | 22.08 |

*Denotes correct answer

*Supplementary Table 53. Players Answers to Q3.11 Do you agree or disagree with these statements on vitamins and minerals? Athletes aged 15 to 24 years need 500 mg of calcium each day*

| **Agree** | | **Disagree*** | | **Not sure** | |
| --- | --- | --- | --- | --- | --- |
| ***n*** | **%** | ***n*** | **%** | ***n*** | **%** |
| 24 | 31.17 | 8 | 10.39 | 45 | 58.44 |

*Denotes correct answer

*Supplementary Table 54. Players Answers to Q3.12 Do you agree or disagree with these statements on vitamins and minerals? A fit person eating a balanced diet can improve their athletic performance by eating more vitamins and minerals from food*

| **Agree** | | **Disagree*** | | **Not sure** | |
| --- | --- | --- | --- | --- | --- |
| ***n*** | **%** | ***n*** | **%** | ***n*** | **%** |
| 56 | 72.73 | 9 | 11.69 | 12 | 15.58 |

*Denotes correct answer

*Supplementary Table 55. Players Answers to Q3.13 Do you agree or disagree with these statements on vitamins and minerals? Vitamins contain energy (kilojoules/calories)*

| **Agree** | | **Disagree*** | | **Not sure** | |
| --- | --- | --- | --- | --- | --- |
| ***n*** | **%** | ***n*** | **%** | ***n*** | **%** |
| 23 | 29.87 | 41 | 53.25 | 13 | 16.88 |

*Denotes correct answer

*Supplementary Table 56. Players Answers to Q4.1 Athletes should drink water to:*

| **Keep plasma (blood) volume stable*** | | **Allow proper sweating** | | **All of the above** | | **Not sure** | |
| --- | --- | --- | --- | --- | --- | --- | --- |
| ***n*** | **%** | ***n*** | **%** | ***n*** | **%** | ***n*** | **%** |
| 9 | 11.69 | 4 | 5.19 | 61 | 79.22 | 3 | 3.90 |

*Denotes correct answer

*Supplementary Table 57. Players Answers to Q4.2 Experts think that athletes should:*

| **Drink 50-100 ml (1.7-3.3 fluid ounces) every 15-20 minutes** | | **Drink sports drinks (e.g. Powerade) rather than water when exercising** | | **Drink to a plan, based on bodyweight changes during training sessions performed in a similar climate*** | | **Not sure** | |
| --- | --- | --- | --- | --- | --- | --- | --- |
| ***n*** | **%** | ***n*** | **%** | ***n*** | **%** | ***n*** | **%** |
| 22 | 28.57 | 3 | 3.90 | 47 | 61.04 | 5 | 6.49 |

*Denotes correct answer

*Supplementary Table 58. Players Answers to Q4.3 How much sodium (salt) should fluid consumed for hydration purposes (during exercise) contain?*

| **Al least 11-25 mmol/L (approx. 250-575 mg/L)*** | | **At least 4-8 mmol/L (approx. 90-185 mg/L)** | | **None** | | **Not sure** | |
| --- | --- | --- | --- | --- | --- | --- | --- |
| ***n*** | **%** | ***n*** | **%** | ***n*** | **%** | ***n*** | **%** |
| 6 | 7.79 | 19 | 24.68 | 6 | 7.79 | 46 | 59.74 |

*Denotes correct answer

*Supplementary Table 59. Players Answers to Q4.4 Before competition, athletes should eat foods that are high in:*

| **Fluids, fats and carbohydrate** | | **Fluids, fibre and carbohydrate** | | **Fluids and carbohydrate*** | | **Not sure** | |
| --- | --- | --- | --- | --- | --- | --- | --- |
| ***n*** | **%** | ***n*** | **%** | ***n*** | **%** | ***n*** | **%** |
| 20 | 25.97 | 15 | 22.08 | 42 | 54.55 | 0 | 0 |

*Denotes correct answer

*Supplementary Table 60. Players Answers to Q4.5 Do you agree or disagree with the statements on carbohydrate? Eating carbohydrates when you exercise makes it harder to build strength and muscles*

| **Agree** | | **Disagree*** | | **Not sure** | |
| --- | --- | --- | --- | --- | --- |
| ***n*** | **%** | ***n*** | **%** | ***n*** | **%** |
| 6 | 7.79 | 63 | 81.82 | 8 | 10.39 |

*Denotes correct answer

*Supplementary Table 61. Players Answers to Q4.6 Do you agree or disagree with the statements on carbohydrate? In events lasting 60 - 90 minutes, 30- 60 g (1.0 - 2.0 ounces) of carbohydrates should be eaten per hour*

| **Agree*** | | **Disagree** | | **Not sure** | |
| --- | --- | --- | --- | --- | --- |
| ***n*** | **%** | ***n*** | **%** | ***n*** | **%** |
| 41 | 53.25 | 11 | 14.29 | 25 | 32.47 |

*Denotes correct answer

*Supplementary Table 62. Players Answers to Q4.7 Do you agree or disagree with the statements on carbohydrate? Eating carbohydrates when you exercise will help keep blood sugar levels stable*

| **Agree*** | | **Disagree** | | **Not sure** | |
| --- | --- | --- | --- | --- | --- |
| ***n*** | **%** | ***n*** | **%** | ***n*** | **%** |
| 48 | 62.34 | 14 | 18.18 | 15 | 19.48 |

*Denotes correct answer

*Supplementary Table 63. Players Answers to Q4.8 Some athletes get a sore stomach if they eat during exercise. What might make stomach pain worse?*

| **Having gels rather than water or sports drinks*** | | **Having small amounts of water at a time** | | **Having sports drinks with different types of carbohydrates (e.g. fructose and sucrose)** | | **Not sure** | |
| --- | --- | --- | --- | --- | --- | --- | --- |
| ***n*** | **%** | ***n*** | **%** | ***n*** | **%** | ***n*** | **%** |
| 27 | 35.06 | 5 | 6.49 | 34 | 44.16 | 11 | 14.29 |

*Denotes correct answer

*Supplementary Table 64. Players Answers to Q4.9 During a competition, athletes should eat foods that are high in:*

| **Fluids, fibre and fat** | | **Fluids and protein** | | **Fluids and carbohydrate*** | | **Not sure** | |
| --- | --- | --- | --- | --- | --- | --- | --- |
| ***n*** | **%** | ***n*** | **%** | ***n*** | **%** | ***n*** | **%** |
| 2 | 2.60 | 7 | 9.09 | 67 | 87.01 | 1 | 1.30 |

*Denotes correct answer

*Supplementary Table 65. Players Answers to Q4.10 Which is the best snack to have during an intense 90-minute training session?*

| **A protein shake** | | **A ripe banana*** | | **A handful of nuts** | | **Not sure** | |
| --- | --- | --- | --- | --- | --- | --- | --- |
| ***n*** | **%** | ***n*** | **%** | ***n*** | **%** | ***n*** | **%** |
| 2 | 2.60 | 70 | 90.91 | 5 | 6.49 | 0 | 0 |

*Denotes correct answer

*Supplementary Table 66. Players Answers to Q4.11 After a competition, athletes should eat foods that are high in?*

| **Protein, carbohydrate and fat** | | **Only protein** | | **Only carbohydrate** | | **Carbohydrate and protein*** | | **Not sure** | |
| --- | --- | --- | --- | --- | --- | --- | --- | --- | --- |
| ***n*** | **%** | ***n*** | **%** | ***n*** | **%** | ***n*** | ***%*** | ***n*** | **%** |
| 30 | 38.96 | 5 | 6.49 | 1 | 1.30 | 41 | 53.25 | 0 | 0 |

*Denotes correct answer

*Supplementary Table 67. Players Answers to Q4.12 How much protein do you think experts say athletes should eat after resistance exercise?*

| **0.3 g/kg bodyweight (approx. 15-25 g [0.53-0.88 ounces) for most athletes*** | | **1.0 g/kg bodyweight (approx. 50-100 [1.9-2.3 ounces) for most athletes** | | **1.5 g/kg bodyweight (approx. 150-230 g [5.3-10.6 ounces) for most athletes** | | **Not sure** | |
| --- | --- | --- | --- | --- | --- | --- | --- |
| ***n*** | **%** | ***n*** | **%** | ***n*** | **%** | ***n*** | **%** |
| 19 | 24.68 | 23 | 29.87 | 22 | 28.57 | 17 | 22.08 |

*Denotes correct answer

*Supplementary Table 68. Players Answers to Q5.1 Do you agree or disagree with the statements about vitamin and mineral supplements? Vitamin C should always be taken by athletes*

| **Agree** | | **Disagree*** | | **Not sure** | |
| --- | --- | --- | --- | --- | --- |
| ***n*** | **%** | ***n*** | **%** | ***n*** | **%** |
| 31 | 40.26 | 37 | 48.05 | 9 | 11.69 |

*Denotes correct answer

*Supplementary Table 69. Players Answers to Q5.2 Do you agree or disagree with the statements about vitamin and mineral supplements? B vitamins should be taken if energy levels are low*

| **Agree** | | **Disagree*** | | **Not sure** | |
| --- | --- | --- | --- | --- | --- |
| ***n*** | **%** | ***n*** | **%** | ***n*** | **%** |
| 32 | 41.56 | 14 | 18.18 | 31 | 40.26 |

*Denotes correct answer

*Supplementary Table 70. Players Answers to Q5.3 Do you agree or disagree with the statements about vitamin and mineral supplements? Salt tablets should be taken by athletes that get cramps when they exercise*

| **Agree** | | **Disagree*** | | **Not sure** | |
| --- | --- | --- | --- | --- | --- |
| ***n*** | **%** | ***n*** | **%** | ***n*** | **%** |
| 41 | 53.25 | 18 | 23.38 | 18 | 23.38 |

*Denotes correct answer

*Supplementary Table 71. Players Answers to Q5.4 Do you agree or disagree with the statements about vitamin and mineral supplements? Iron tablets should be taken by all athletes who feel tired and are pale*

| **Agree** | | **Disagree*** | | **Not sure** | |
| --- | --- | --- | --- | --- | --- |
| ***n*** | **%** | ***n*** | **%** | ***n*** | **%** |
| 40 | 51.95 | 24 | 31.17 | 13 | 16.88 |

*Denotes correct answer

*Supplementary Table 72. Players Answers to Q5.5 All supplements are tested to make sure they are safe, don’t have any contamination.*

| **Agree** | | **Disagree*** | | **Not sure** | |
| --- | --- | --- | --- | --- | --- |
| ***n*** | **%** | ***n*** | **%** | ***n*** | **%** |
| 18 | 23.38 | 55 | 71.43 | 4 | 5.19 |

*Denotes correct answer

*Supplementary Table 73. Players Answers to Q5.6 Supplement labels may sometimes say things that are not true.*

| **Agree*** | | **Disagree** | | **Not sure** | |
| --- | --- | --- | --- | --- | --- |
| ***n*** | **%** | ***n*** | **%** | ***n*** | **%** |
| 65 | 84.42 | 8 | 10.39 | 4 | 5.19 |

*Denotes correct answer

*Supplementary Table 74. Players Answers to Q5.7 Do you agree or disagree with the statements about supplements? Creatine makes the brain think that exercise feels easier*

| **Agree** | | **Disagree*** | | **Not sure** | |
| --- | --- | --- | --- | --- | --- |
| ***n*** | **%** | ***n*** | **%** | ***n*** | **%** |
| 9 | 11.69 | 44 | 57.14 | 24 | 31.17 |

*Denotes correct answer

*Supplementary Table 75. Players Answers to Q5.8 Do you agree or disagree with the statements about supplements? Caffeine makes muscles able to work harder even without more oxygen*

| **Agree** | | **Disagree*** | | **Not sure** | |
| --- | --- | --- | --- | --- | --- |
| ***n*** | **%** | ***n*** | **%** | ***n*** | **%** |
| 13 | 16.88 | 45 | 58.44 | 19 | 24.68 |

*Denotes correct answer

*Supplementary Table 76. Players Answers to Q5.9 Do you agree or disagree with the statements about supplements? Beetroot juice (nitrates) makes muscles feel less sore after exercise*

| **Agree** | | **Disagree*** | | **Not sure** | |
| --- | --- | --- | --- | --- | --- |
| ***n*** | **%** | ***n*** | **%** | ***n*** | **%** |
| 39 | 50.65 | 12 | 15.58 | 26 | 33.77 |

*Denotes correct answer

*Supplementary Table 77. Players Answers to Q5.10 Do you agree or disagree with the statements about supplements? Beta-Alanine can decrease how much acid muscles make during intense exercise*

| **Agree*** | | **Disagree** | | **Not sure** | |
| --- | --- | --- | --- | --- | --- |
| ***n*** | **%** | ***n*** | **%** | ***n*** | **%** |
| 21 | 27.27 | 8 | 10.39 | 48 | 62.34 |

*Denotes correct answer

*Supplementary Table 78. Players Answers to Q5.11 Which supplement does not have enough evidence in relation to improving body composition or sporting performance?*

| **Caffeine** | | **Ferulic acid*** | | **Bicarbonate** | | **Leucine** | | **Not sure** | |
| --- | --- | --- | --- | --- | --- | --- | --- | --- | --- |
| ***n*** | **%** | ***n*** | **%** | ***n*** | **%** | ***n*** | ***%*** | ***n*** | **%** |
| 12 | 15.58 | 8 | 10.39 | 11 | 14.29 | 2 | 2.60 | 44 | 57.14 |

*Denotes correct answer

*Supplementary Table 79. Players Answers to Q5.12 WORLD ANTI-DOPING AGENCY (WADA) bans the use of….*

| **Caffeine** | | **Bicarbonate** | | **Carnitine** | | **Testosterone*** | | **Not sure** | |
| --- | --- | --- | --- | --- | --- | --- | --- | --- | --- |
| ***n*** | **%** | ***n*** | **%** | ***n*** | **%** | ***n*** | ***%*** | ***n*** | **%** |
| 0 | 0 | 0 | 0 | 3 | 3.90 | 69 | 76.62 | 5 | 6.49 |

*Denotes correct answer

*Supplementary Table 80. Players Answers to Q6.1 How much ethanol (pure alcohol) is there in a standard drink?*

| **1-2 g / 0.03-0.06 fluid ounces** | | **8-14 g / 0.3-0.6 fluid ounces*** | | **30-50 g / 1.2-2.0fluid ounces** | | **Not sure** | |
| --- | --- | --- | --- | --- | --- | --- | --- |
| ***n*** | **%** | ***n*** | **%** | ***n*** | **%** | ***n*** | **%** |
| 12 | 15.58 | 34 | 44.16 | 2 | 2.60 | 29 | 37.66 |

*Denotes correct answer

*Supplementary Table 81. Players Answers to Q6.2 Which is an example of a "Standard Drink"?*

| **30-45 ml / 1-1.5 fluid ounces of pure spirits*** | | **One quarter of a bottle (175 ml / 6 fluid ounces) of red wine** | | **A pint (425 ml / 14 fluid ounces) of full-strength beer** | | **Not sure** | |
| --- | --- | --- | --- | --- | --- | --- | --- |
| ***n*** | **%** | ***n*** | **%** | ***n*** | **%** | ***n*** | **%** |
| 21 | 27.27 | 14 | 18.18 | 29 | 37.66 | 13 | 16.88 |

*Denotes correct answer

*Supplementary Table 82. Players Answers to Q6.3 Do you think alcohol can make you put on weight?*

| **Yes*** | | **No** | | **Not sure** | |
| --- | --- | --- | --- | --- | --- |
| ***n*** | **%** | ***n*** | **%** | ***n*** | **%** |
| 75 | 97.40 | 2 | 2.60 | 0 | 0 |

*Denotes correct answer

*Supplementary Table 83. Players Answers to Q6.4 How many drinks do you think experts say are the most we should have in one day?*

| **Two*** | | **Three** | | **Four** | | **Not sure** | |
| --- | --- | --- | --- | --- | --- | --- | --- |
| ***n*** | **%** | ***n*** | **%** | ***n*** | **%** | ***n*** | **%** |
| 53 | 68.83 | 7 | 9.09 | 5 | 6.49 | 12 | 15.58 |

*Denotes correct answer

*Supplementary Table 84. Players Answers to Q6.5 Do you agree or disagree with the statements on alcohol? If someone does not drink at all during the week, it is okay for them to have five or more drinks on a Friday or Saturday night*

| **Agree** | | **Disagree*** | | **Not sure** | |
| --- | --- | --- | --- | --- | --- |
| ***n*** | **%** | ***n*** | **%** | ***n*** | **%** |
| 7 | 9.09 | 68 | 88.31 | 2 | 2.60 |

*Denotes correct answer

*Supplementary Table 85. Players Answers to Q6.6 Do you agree or disagree with the statements on alcohol? Drinking lots of alcohol can make it harder to recover from injury*

| **Agree*** | | **Disagree** | | **Not sure** | |
| --- | --- | --- | --- | --- | --- |
| ***n*** | **%** | ***n*** | **%** | ***n*** | **%** |
| 67 | 87.01 | 3 | 3.90 | 7 | 9.09 |

*Denotes correct answer

*Supplementary Table 86. Players Answers to Q6.7 Do you agree or disagree with the statements on alcohol? Alcohol makes you urinate more*

| **Agree*** | | **Disagree** | | **Not sure** | |
| --- | --- | --- | --- | --- | --- |
| ***n*** | **%** | ***n*** | **%** | ***n*** | **%** |
| 57 | 74.03 | 11 | 14.29 | 9 | 11.69 |

*Denotes correct answer

*Supplementary Table 87. Players Answers to Q6.8 "Binge drinking" (also referred to as heavy episodic drinking) is defined as:*

| **Having two or more standard alcoholic drinks on the same occasion** | | **Having four to five standard alcoholic drinks on the same occasion*** | | **Having seven to eight standard alcoholic drinks on the same occasion** | | **Not sure** | |
| --- | --- | --- | --- | --- | --- | --- | --- |
| ***n*** | **%** | ***n*** | **%** | ***n*** | **%** | ***n*** | **%** |
| 6 | 7.79 | 39 | 50.65 | 25 | 32.47 | 7 | 9.09 |

*Denotes correct answer
